# Biological and Clinical Factors Contributing to the Metabolic Heterogeneity of Hospitalized Patients with and without COVID-19

**DOI:** 10.3390/cells10092293

**Published:** 2021-09-02

**Authors:** Angelo D’Alessandro, Tiffany Thomas, Imo J. Akpan, Julie A. Reisz, Francesca I. Cendali, Fabia Gamboni, Travis Nemkov, Kiruphagaran Thangaraju, Upendra Katneni, Kenichi Tanaka, Stacie Kahn, Alexander Z. Wei, Jacob E. Valk, Krystalyn E. Hudson, David Roh, Chiara Moriconi, James C. Zimring, Eldad A. Hod, Steven L. Spitalnik, Paul W. Buehler, Richard O. Francis

**Affiliations:** 1 Department of Biochemistry and Molecular Genetics, Anschutz Medical Campus, University of Colorado Denver, Aurora, CO 80045, USA; julie.haines@ucdenver.edu (J.A.R.); francesca.cendali@cuanschutz.edu (F.I.C.); fabia.gamboni@cuanschutz.edu (F.G.); travis.nemkov@cuanschutz.edu (T.N.); 2 Department of Pathology & Cell Biology, Columbia University Vagelos College of Physicians and Surgeons, New York, NY 10032, USA; tt2254@cumc.columbia.edu (T.T.); jv2671@cumc.columbia.edu (J.E.V.); keh2197@cumc.columbia.edu (K.E.H.); cm3952@cumc.columbia.edu (C.M.); eh2217@cumc.columbia.edu (E.A.H.); ss2479@cumc.columbia.edu (S.L.S.); rof3@cumc.columbia.edu (R.O.F.); 3 Division of Hematology/Oncology, Department of Medicine, Irving Medical Center, Columbia University, New York, NY 10032, USA; ija2117@cumc.columbia.edu (I.J.A.); sck9012@nyp.org (S.K.); aw3056@cumc.columbia.edu (A.Z.W.); 4 Center for Blood Oxygen Transport, Department of Pathology, Department of Pediatrics, University of Maryland, Baltimore, MD 21201, USA; KThangaraju@som.umaryland.edu (K.T.); UKatneni@som.umaryland.edu (U.K.); pbuehler@som.umaryland.edu (P.W.B.); 5 Department of Anesthesiology, University of Maryland, Baltimore, MD 21201, USA; Kenichi-Tanaka@ouhsc.edu; 6 Department of Anesthesiology, University of Oklahoma College of Medicine, Oklahoma City, OK 73126-0901, USA; 7 Department of Neurology, Columbia University Vagelos College of Physicians and Surgeons, New York, NY 10032, USA; dr2753@cumc.columbia.edu; 8 Department of Pathology, University of Virginia, Charlottesville, VA 22903, USA; jcz2k@virginia.edu

**Keywords:** COVID-19, metabolomics, tryptophan, kynurenine, amino acid, fatty acid, acylcarnitine

## Abstract

The Corona Virus Disease 2019 (COVID-19) pandemic represents an ongoing worldwide challenge. The present large study sought to understand independent and overlapping metabolic features of samples from acutely ill patients (n = 831) that tested positive (n = 543) or negative (n = 288) for COVID-19. High-throughput metabolomics analyses were complemented with antigen and enzymatic activity assays on plasma from acutely ill patients collected while in the emergency department, at admission, or during hospitalization. Lipidomics analyses were also performed on COVID-19-positive or -negative subjects with the lowest and highest body mass index (n = 60/group). Significant changes in amino acid and fatty acid/acylcarnitine metabolism emerged as highly relevant markers of disease severity, progression, and prognosis as a function of biological and clinical variables in these patients. Further, machine learning models were trained by entering all metabolomics and clinical data from half of the COVID-19 patient cohort and then tested on the other half, yielding ~78% prediction accuracy. Finally, the extensive amount of information accumulated in this large, prospective, observational study provides a foundation for mechanistic follow-up studies and data sharing opportunities, which will advance our understanding of the characteristics of the plasma metabolism in COVID-19 and other acute critical illnesses.

## 1. Introduction

On 28 April 2020, we began performing one of the earliest investigations on the impact of SARS-CoV-2 infection on the circulating metabolome [1]. At that time, we reported that ~3 million cases had been confirmed worldwide, a number that has dramatically risen since to ~200 million cases and 4 million deaths—according to the World Health Organization (https://www.who.int/emergencies/diseases/novel-coronavirus-2019—accessed on 10 May 2021). Our original study aimed to identify metabolic signatures that could help inform prognosis and guide treatment early after the onset of COVID-19, the disease caused by SARS-CoV-2 infection. Indeed, small-molecule metabolites provide the building blocks that fuel viral replication, from nucleic acids to proteins and membrane lipids. SARS-CoV-2 [1,2,3,4,5], like other viral infections [6], was found to promote the mobilization of free fatty acids to support the formation of viral capsid-associated membranes; a phenomenon that could be explained, at least in part, by activation of phospholipase A2 [7,8], a target amenable to pharmacological intervention.

Despite public health interventions and the advent of multiple vaccination strategies, SARS-CoV-2 remains a serious global threat. This may be explained by multiple factors, including (i) vaccination rates lagging behind the percentage required to reach herd immunity; (ii) reopening too early, while discontinuing public health mandates; (iii) the emergence of variants with more efficient transmission [9,10], which may also escape acquired immunity and/or vaccination [11,12]; and waning immunity from vaccination or previous infection [13,14]. Therefore, efforts aimed at identifying strategies to treat SARS-CoV-2, including metabolic interventions or repurposing drugs with potential metabolic targets [15], remain important.

To this end, herein we prospectively collected a large independent data set, including patient characteristics, clinical information, and routine and specialized clinical laboratory results of acutely ill patients that were admitted to one of two large university hospitals in New York City, who tested positive or negative for SARS-CoV-2. This large patient group allowed for the verification and expansion of previously published metabolomics studies in COVID-19 and other acute critical illnesses. 

## 2. Materials and Methods

***Patients***: This study was approved by the Institutional Review Board of Columbia University Irving Medical Center (CUIMC) (Protocol Number AAAT0680). The laboratory information system (Cerner Millennium) was used to identify all patients from 1 March 2020 through 31 May 2020 who had antithrombin (AT) testing ordered. It was then determined if these patients had been tested for the presence of SARS-CoV-2 based upon the availability of SARS-CoV-2 PCR results in the patient’s electronic medical record. All patients with AT and SARS-CoV-2 testing during the aforementioned 3-month time window were included in this study. The frozen, residual, discarded sample from AT testing was used for metabolomics and lipidomics analysis. 

Data were obtained from 831 patients who were admitted or seen in the Emergency Department from 14 April 2020 through 31 May 2020 in one of two large hospitals in New York City (i.e., before the identification of and routine testing for novel variants in the USA). The patients were stratified based on SARS-CoV-2 RT-PCR positivity status (n = 543 SARS-CoV-2 positive; n = 288 SARS-CoV-2 negative). 

As part of routine care, hemostasis was evaluated on STAR Evolution and STAR Max analyzers (Diagnostica Stago, Parsippany, NJ, USA), hematology testing by Sysmex XN900 (Lincolnshire, IL, USA), and chemistry testing by Roche Cobas c502 (Indianapolis, IN). Laboratory values, including antithrombin (AT), prothrombin time (PT)/international normalized ratio (INR), activated partial thromboplastin time aPTT, fibrinogen, d-dimer, white blood cell count (WBC), absolute neutrophil count (ANC), absolute lymphocyte count (ALC), absolute monocyte count (AMC), hemoglobin, red blood cell count (RBC), RBC distribution width (RDW), reticulocyte count, platelet count, IL-6, lactate dehydrogenase (LDH), lactic acid, procalcitonin, troponin, blood urea nitrogen (BUN), creatinine, glucose, total-, direct-, and indirect bilirubin, aspartate amino transferase (AST), alanine amino transferase (ALT), albumin, total protein, ferritin, C-reactive protein (CRP), erythrocyte sedimentation rate (ESR), creatine kinase (CK), triglycerides, and blood type, were collected. 

Laboratory data were obtained from the Clinical Data Warehouse at Columbia University Irving Medical Center (CUIMC) after approval from the Tripartite Request Assessment Committee. Clinical and demographic data, including sex, age, race, ethnicity, weight, body mass index, comorbidities (hypertension, diabetes mellitus, coronary artery disease, renal disease, hyperlipidemia, liver disease, lung disease), intubation/ventilator requirement, continuous veno-venous hemofiltration (CVVH) requirement, radiographically confirmed thrombotic complications (deep vein thrombosis, pulmonary embolism, stroke), clotting of CVVH, hospitalization course (admission date, date of Emergency Department presentation, discharge date), mortality, and date of death were collected manually by reviewing the electronic medical record. Data were collected retrospectively for patients treated at two New York Presbyterian Hospital campuses (CUIMC and The Allen hospital). Residual platelet-poor plasma samples were collected for subsequent analyses.

***Sample processing and metabolite extraction*:** Plasma samples were extracted via a modified Folch method (chloroform/methanol/water 8:4:3 *v*/*v*/*v*), which completely inactivates other coronaviruses, such as MERS-CoV [16]. Briefly, 20 uL of plasma were diluted in 130 uL of LC-MS grade water, 600 uL of ice-cold chloroform/methanol (2:1) was added, and the samples vortexed for 10 s. Samples were then incubated at 4 °C for 5 min, quickly vortexed (5 s), and centrifuged at 14,000× g for 10 min at 4 °C. The top (i.e., aqueous) phase was transferred to a new tube for metabolomics analysis. 

***Ultra-High-Pressure Liquid Chromatography–Mass Spectrometry metabolomics and lipidomics***: Analyses were performed using a Vanquish UHPLC coupled online to a Q Exactive mass spectrometer (Thermo Fisher, Bremen, Germany). Samples were analyzed using a 5 and 17 min gradient as described [17,18,19]. Solvents were supplemented with 0.1% formic acid for positive mode runs and 1 mM ammonium acetate for negative mode runs. MS acquisition and data analysis was performed as previously described [17,18,19]. 

***Metabolomics***: UHPLC-MS metabolomics analyses were performed as described in method [17,18,19] and application papers [1,20], using a Vanquish UHPLC system coupled online to a high-resolution Q Exactive mass spectrometer (Thermo Fisher, Bremen, Germany). Samples were resolved over a Kinetex C18 column (2.1 × 150 mm^2^, 1.7 µm; Phenomenex, Torrance, CA, USA) at 45°C. A volume of 10 uL of sample extracts from the aqueous phase of the Folch extraction was injected into the UHPLC-MS. Each sample was injected and run four times with two different chromatographic and MS conditions as follows: (1) using a 5 min gradient at 450 µL/minute from 5–95% B (A: water/0.1% formic acid; B: acetonitrile/0.1% formic acid) and the MS was operated in positive mode and (2) using a 5 min gradient at 450 µL/minute from 5–95% B (A: 5% acetonitrile, 95%water/1 mM ammonium acetate; B:95%acetonitrile/5% water, 1 mM ammonium acetate) and the MS was operated in negative ion mode. The UHPLC system was coupled online with a Q Exactive (Thermo, San Jose, CA, USA) scanning in Full MS mode at 70,000 resolution in the 60–900 *m*/*z* range, 4 kV spray voltage, 15 sheath gas, and 5 auxiliary gas, operated in negative or positive ion mode (separate runs). These chromatographic and MS conditions were applied for both relative and targeted quantitative metabolomics measurements, with the differences that for the latter targeted quantitative post hoc analyses were performed on the basis of the stable isotope-labeled internal standards used as a reference quantitative measurement, as detailed below.

***Lipidomics***: Samples were resolved as described, 4–6, [21] over an ACQUITY HSS T3 column (2.1 × 150 mm^2^, 1.8 µm particle size (Waters, MA, USA) using an aqueous phase (**A**) of 25% acetonitrile and 5 mM ammonium acetate and a mobile phase (**B**) of 50% isopropanol, 45% acetonitrile, and 5 mM ammonium acetate. Samples were eluted from the column using either the solvent gradient: 0–1 min 25% B and 0.3 mL/min, 1–2 min 25–50% B and 0.3 mL/min, 2–8 min 50–90% B and 0.3 mL/min, 8–10 min 90–99% B and 0.3 mL/min, 10–14 min hold at 99% B and 0.3 mL/min, 14–14.1 min 99–25% B and 0.3 mL/min, 14.1–16.9 min hold at 25% B and 0.4 mL/min, 16.9–17 min hold at 25% B and resume flow of 0.3 mL/min isocratic elution of 5% B flowed at 250 µL/min and 25 °C or a gradient from 0–5% B over 0.5 min, 5–95% B over 0.6 min, hold at 95% B for 1.65 min, 95–5% B over 0.25 min, hold at 5% B for 2 min, flowed at 450 µL/min and 35 °C [19]. The Q Exactive mass spectrometer (Thermo Fisher Scientific, San Jose, CA, USA) was operated independently in positive or negative ion mode, scanning in Full MS mode (2 μscans) from 150 to 1500 *m*/*z* at 70,000 resolution, with 4 kV spray voltage, 45 sheath gas, and 15 auxiliary gas. 

***MS2 analyses for untargeted metabolomics***: For discovery mode untargeted metabolomics, dd-MS2 was performed at 17,500 resolution, AGC target = 1 × 10^5^, maximum IT = 50 ms, and stepped NCE of 25, 35 for positive mode, and 20, 24, and 28 for negative mode, as described in Stefeanoni et al. [22], and applied to similar samples (i.e., stored RBCs) in D’Alessandro et al. [23].

***Quality control and data processing***: Calibration was performed prior to analysis using the Pierce ESI Positive and Negative Ion Calibration Solutions (Thermo Fisher Scientific). Acquired data were then converted from raw to .mzXML file format using Mass Matrix (Cleveland, OH, USA). Samples were analyzed in randomized order with a technical mixture (generated by mixing 5 uL of all samples tested in this study) injected every 10 runs to qualify instrument performance. This technical mixture was also injected three times per polarity mode and analyzed with the parameters above, except CID fragmentation was included for unknown compound identification (10 ppm error for both positive and negative ion mode searches for intact mass, 50 ppm error tolerance for fragments in MS2 analyses—further details about the database searched below).

***Metabolite assignment and relative quantitation***: Metabolite assignments, isotopologue distributions, and correction for expected natural abundances of deuterium, 13C, and 15N isotopes were performed using MAVEN (Princeton, NJ, USA) [24], against an in-house library of deuterated lipid standards (SPLASH^®®^ LIPIDOMIX^®®^ Mass Spec Standard, Avanti Lipids) and in-house libraries of 3000 unlabeled (IROATech product A2574 by ApexBio MSMLS, IROATech, Bolton, MA, USA; standard compounds for central carbon and nitrogen pathways from SIGMA Aldrich, St Louis, MO, USA) and labeled standards (see below for the latter). Discovery mode analysis was performed with standard workflows using Compound Discoverer 2.1 SP1 (Thermo Fisher Scientific, San Jose, CA, USA). From these analyses, metabolite IDs or unique chemical formulae were determined from high-resolution accurate intact mass, isotopic patterns, identification of eventual adducts (e.g., Na+ or K+, etc.) and MS2 fragmentation spectra against the KEGG pathway, HMDB, ChEBI, and ChEMBL databases. Additional untargeted lipidomics analyses were performed with the software LipidSearch (Thermo Fisher, Bremen, Germany). 

***Simultaneous thrombin and plasmin generation assay (STPGA*)**: Simultaneous evaluation of thrombin and plasmin generation (TG and PG, respectively) was performed as described previously [25]. Briefly, plasma samples were mixed with either thrombin specific substrate, Z-Gly-Gly-Arg-AMC (Bachem, Bubendorf, Switzerland) or plasmin specific substrate, Boc-Glu-Lys-Lys-AMC (Bubendorf, Switzerland), and 16 nM of thrombomodulin (PeproTech, Rocky Hill, NJ, USA). The reaction was initiated by adding an activator solution that yielded a final concentration of 1 pM tissue factor (Diagnostica Stago, Parsippany, NJ, USA), 0.7 µg/mL of tissue plasminogen activator (Sigma-Aldrich, St. Louis, MO, USA), and 16 mM CaCl2. Sample wells supplemented with buffer (150 mM NaCl and 20 mM HEPES) and AMC fluorophore instead of activator solution were used for background and calibrator measurements, respectively. Calculation of thrombin and plasmin concentration was performed as described previously [26]

***VWF, FVIII, and ADAMTS13 activity and antigen quantitation***: The antigen and activity measurement of VWF and ADAMTS13 was performed by using commercial ELISA kits. VWF antigen and collagen-binding activity levels were measured by using Human Von Willebrand Factor ELISA Kit (ab168548, Abcam, Cambridge, UK) and TECHNOZYM^®®^ vWF:CBA ELISA Kit (5450301, Technoclone, Vienna, Austria), respectively. ADAMTS13 antigen and activity levels were measured by using Human ADAMTS13 ELISA Kit (ab234559, Abcam) and TECHNOZYM^®®^ ADAMTS13 Activity ELISA (5450701, Technoclone), respectively. FVIII antigen levels were measured by using Human Factor VIII total antigen assay ELISA kit (HFVIIIKT-TOT, Molecular Innovations, Novi, MI, USA). All assays were performed following manufacturer’s recommendations with additional dilution of plasma samples as required.

***Statistical Analysis***: Graphs and statistical analyses (either t-test or repeated measures ANOVA) were prepared with GraphPad Prism 5.0 (GraphPad Software, Inc, La Jolla, CA, USA), GENE E (Broad Institute, Cambridge, MA, USA), and MetaboAnalyst 4.0. In MetaboAnalyst, relative quant data (but not for abs quant), raw values for integrated peak areas for each metabolite, were normalized on a pool of day 0 controls and auto-scaled for each species independently prior to margining all the data for multivariate analysis. Analyses through MetaboAnalyst included principal component analysis, partial least square discriminant analysis, hierarchical clustering analyses (including time-series repeated measures and two-way ANOVA analyses), calculation of receiver operating characteristic (ROC) curves, correlation analyses (Spearman), and machine learning analyses (random forest, support-vector machine—SVM). 

## 3. Results

### 3.1. COVID-19 Patients Display Significant Markers of Kidney Injury, Including Increases in Creatinine and Purine Oxidation, and Decreases in Amino Acids

**Figure 1:**
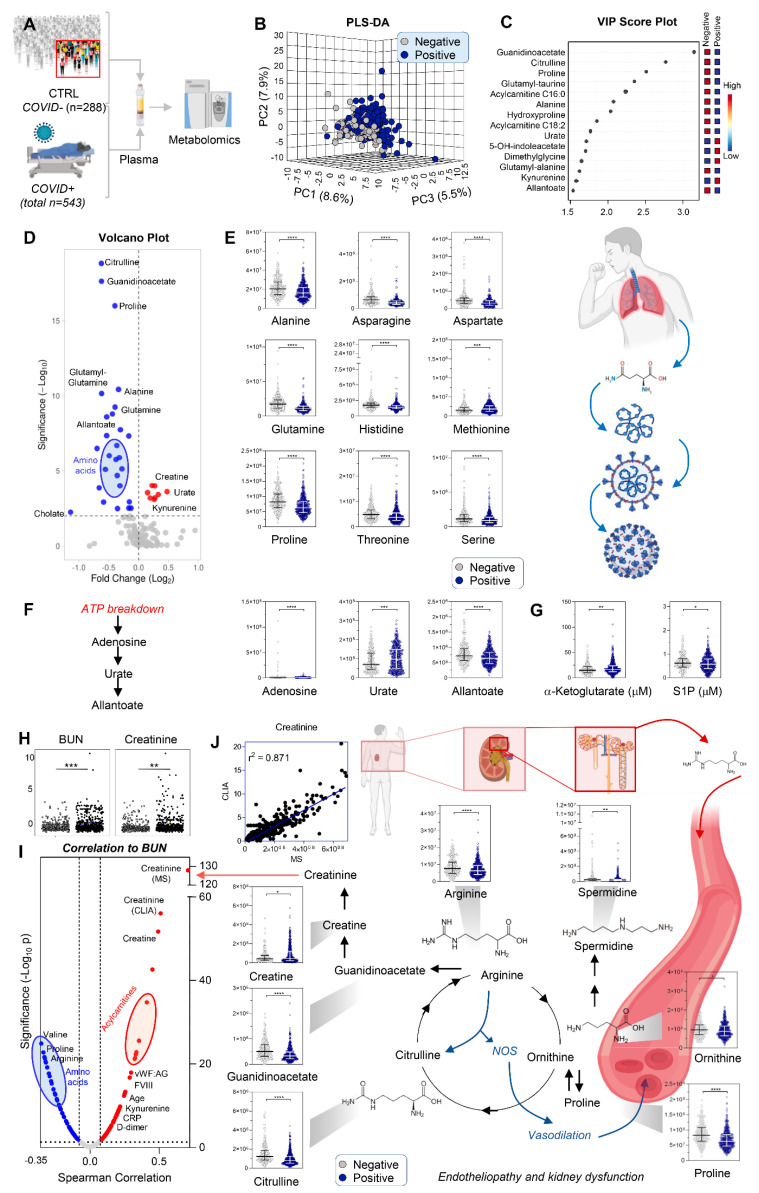
Metabolomics of hospitalized patients with (n = 543) and without (n = 288) COVID-19 (**A**). Partial least square-discriminant analysis of metabolomics data separated the two cohorts (**B**). Top 15 metabolites with the highest loading weights are indicated in the variable importance in projection (VIP) ranked list in (**C**). In (**D**), the volcano plot highlights significant effects of COVID-19 on plasma amino acid levels and purine oxidation. Violin plots (including median + ranges) are shown for amino acids (**E**) and purines (**F**) from relative quantitative analyses, and for two markers of mitochondrial dysfunction and hypoxia, alpha-ketoglutarate and sphingosine 1-phosphate (S1P), using absolute quantitative analyses against stable isotope-labeled internal standards in (**G**). In (**H**), blood urea nitrogen (BUN) and creatinine, markers of kidney dysfunction, were significantly increased in COVID-19 patients. Metabolic and clinical correlates of BUN (top positive correlate being creatinine) are in (**I**). A significant positive correlation (*p* < 0.0001; r2 = 0.871) was observed between creatinine measurements via CLIA-certified and mass spectrometry (MS)-based approaches (**J**). In (**J**), violin plots highlight metabolites in the arginine, proline, and creatine metabolism. Asterisks indicate significance (* *p* < 0.05; ** *p* < 0.01; *** *p* < 0.001; **** *p* < 0.0001).

Metabolomics analyses performed for 543 samples from hospitalized acutely ill COVID-19-positive patients were compared with those performed for 288 samples from acutely ill COVID-19-negative patients (Figure 1A). Raw data, along with clinical characteristics are detailed in Supplementary Table S1; a visual representation of technical mixes as a quality control against test samples confirmed good reproducibility (CV < 20%) in the T-distributed stochastic neighbor embedding (tSNE) analysis (Supplementary Figure S1). 

The metabolic phenotypes of these COVID-19-negative patients partially overlapped with those who were COVID-19-positive (Figure 1B). Figure 1C shows which metabolites differentiate between these two groups, using variable importance in projection (VIP) scores in the partial least squares discriminant analysis (PLS-DA). Volcano plot elaborations (Figure 1D) also clearly showed decreased levels of almost all amino acids with the exception of methionine (Figure 1E) as well as increased levels of purine oxidation products (urate and allantoate) and decreased adenosine (Figure 1F) in COVID-19 patients. Several of these observations were validated using stable isotope-labeled internal standards for absolute quantification (Supplementary Table S1), including markers of hypoxia [27,28], the carboxylic acid alpha-ketoglutarate, and sphingosine 1-phosphate (S1P) (Figure 1G). Notably, amino acid reabsorption occurs in the kidney [29] and alterations in purine metabolism [30] and S1P [31] were recently tied to kidney ischemia and chronic kidney disease, respectively. In addition, moderate–severe kidney dysfunction was observed in all COVID-19 (+) patients, indicated by blood urea nitrogen (BUN) and creatinine levels (Figure 1H). The positive correlation between BUN and creatinine was paralleled by similar trends for acylcarnitines (markers of mitochondrial dysfunction), and negative correlations between BUN and amino acids. As an internal validation of this approach, creatinine measured in the same samples by a CLIA-certified clinical chemistry assay and mass spectrometry correlated extremely well (*p* < 0.0001; r^2^ = 0.871 Spearman; Figure 1J). 

**Figure 2:**
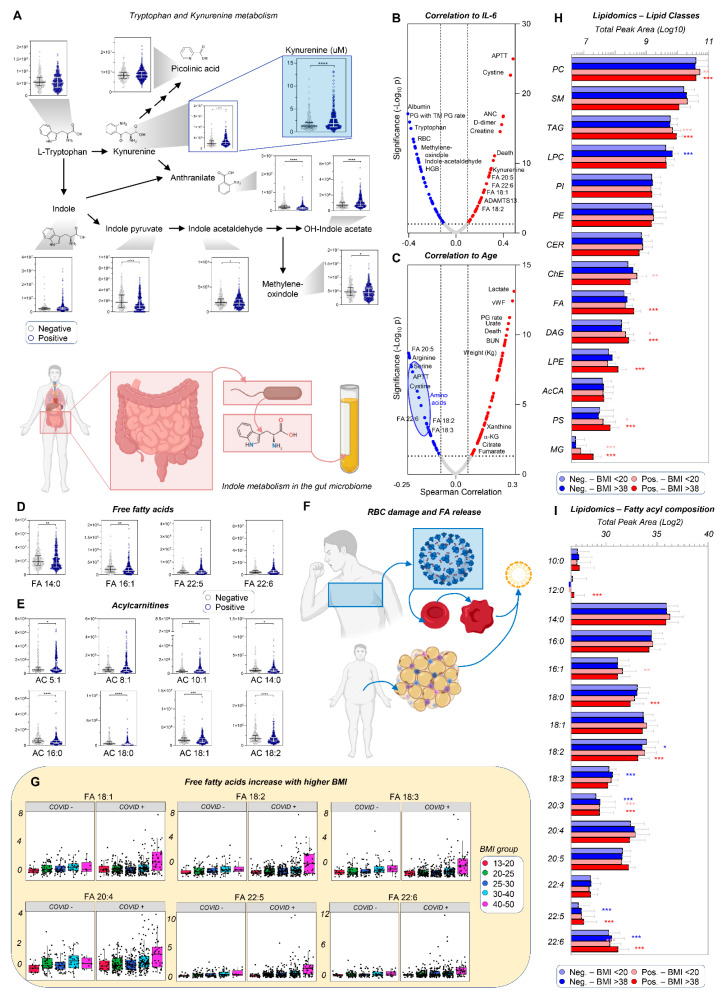
Alteration of tryptophan/kynurenine/indole metabolism as a function of inflammation, and dysregulation of lipid metabolism as a function of body mass index in hospitalized patients with and without COVID-19. In (**A**), violin plot of tryptophan metabolism as a function of COVID status (median + range). Metabolic and clinical correlates to interleukin 6 (IL-6) as a marker of inflammation (**B**) and patient age (**C**) indicate a strong correlation of this pathway and lipid metabolism, especially free fatty acids (**D**) and acylcarnitines (**E**), with disease state. Free fatty acids may derive from blood cell vesiculation and/or mobilization from white adipose tissue, a process that could fuel viral membrane formation (**F**). In (**G**), breakdown of free fatty acid levels as a function of patients’ body mass index (BMI) and COVID-19 status. Lipidomics analyses of COVID-19-positive and -negative patients with BMI lower than 20 or higher than 38 revealed a significant impact of these variables on lipid class (**H**) and fatty acyl composition (**I**). (*p* < 0.05; * *p* < 0.01; ** *p* < 0.001; *** *p* < 0.0001; **** *p* < 0.0001).

**Figure 3:**
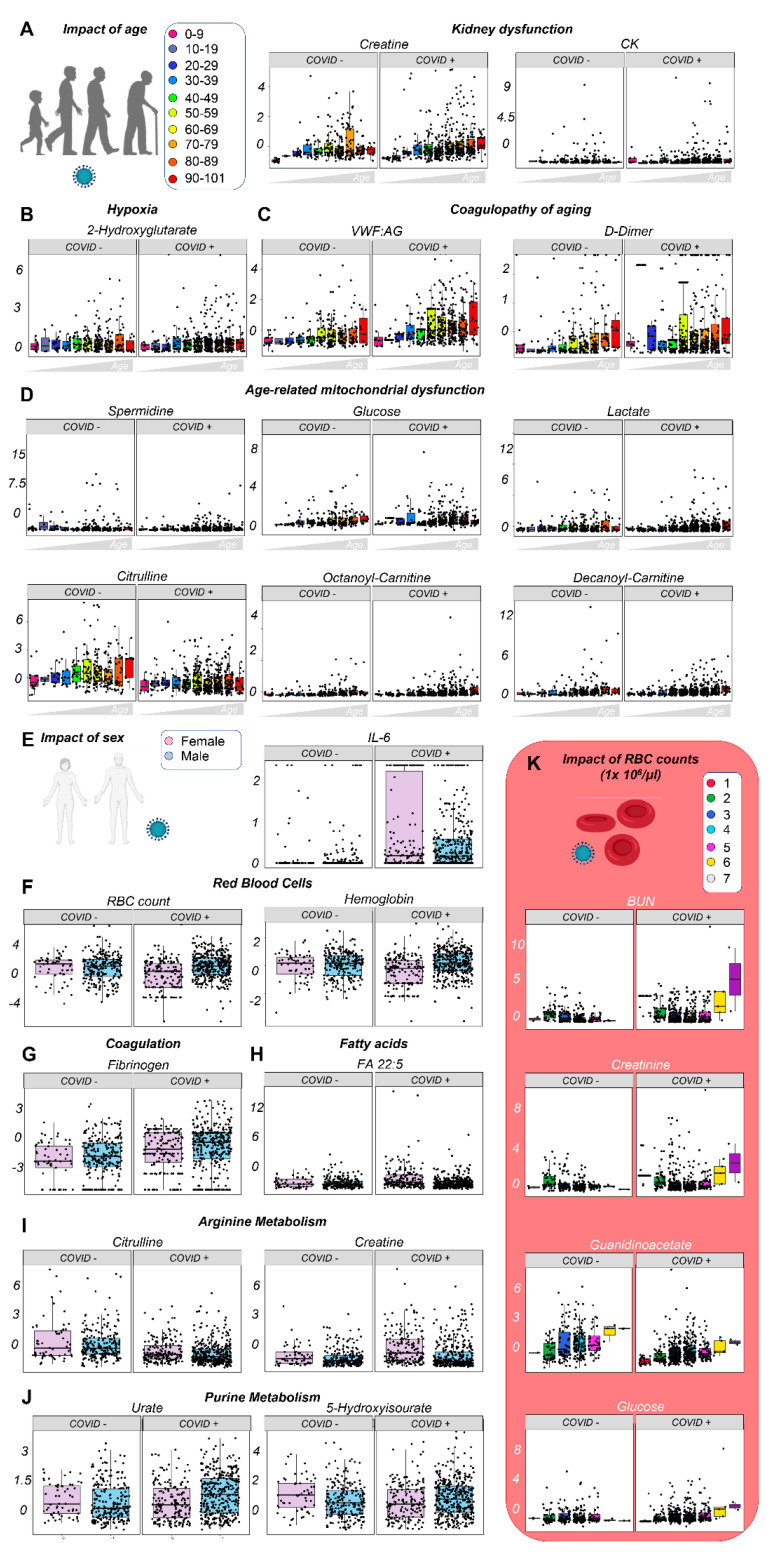
The impact of age and sex on the plasma metabolome of hospitalized patients with or without COVID-19. Patients were clustered into groups depending on their age (**A**). Significant correlates to age or COVID-19 status were identified through Spearman correlation and two-way ANOVA, with top variables including markers of kidney dysfunction (**A**), hypoxia (**B**), coagulopathy (**C**), and age-related mitochondrial dysfunction (**D**). Similar analyses were performed as a function of patients’ COVID-19 status and sex (**E**), with inflammatory markers being significantly affected by COVID-19, and RBC (**F**) and coagulation parameters (**G**) by sex. Similarly, sex affected fatty acid levels (especially poly- and highly unsaturated, long-chain fatty acids), and arginine and purine metabolism (**H**–**J**). Because of the impact of sex on RBC-related parameters, additional analyses were performed highlighting correlates to RBC counts and COVID-19 status, demonstrating a strong correlation with kidney dysfunction (**K**). All the metabolites shown in this figure as dot plots are significant by two-way ANOVA (FDR < 0.05).

**Figure 4:**
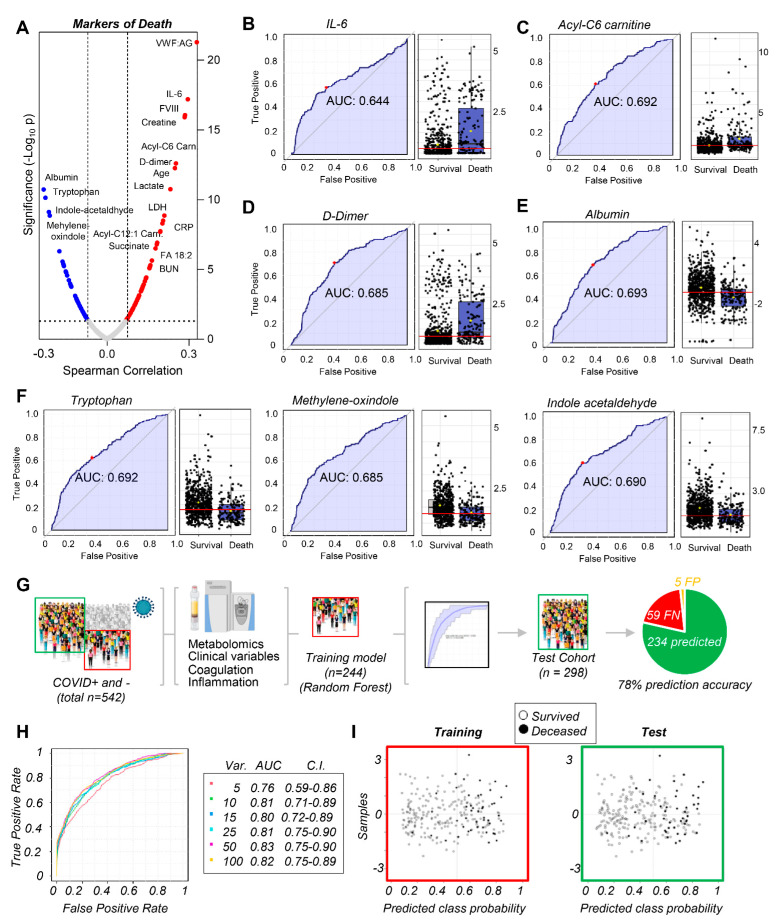
Markers of mortality in hospitalized patients, including COVID-19 patients. In (**A**), clinical and metabolic markers of mortality were ranked from Spearman correlation analyses (y axes indicate −log10 of *p*-values). Because mortality is a non-continuous variable, additional univariate (**B**–**F**) and multivariate (**G**) biomarker analyses were performed to calculate ROC curves and train machine learning algorithms (random forest in this figure, supporting vector machine in the Supplement) to predict mortality in hospitalized COVID-19 patients based on the top 10 clinical and metabolic variables (**H**), a model that yielded 78% prediction accuracy (**G**–**I**).

**Figure 5:**
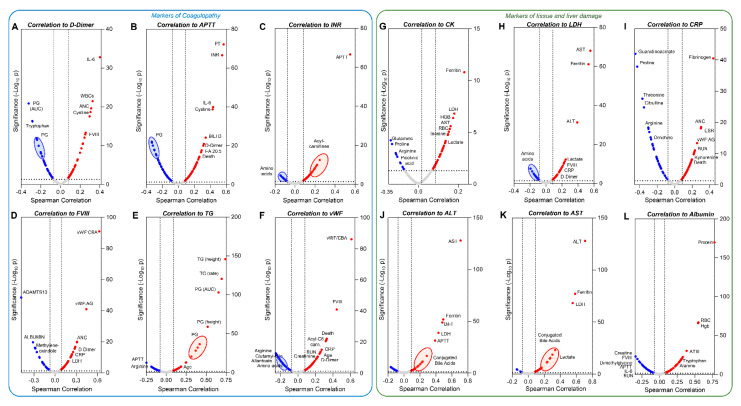
Metabolic correlates to coagulation parameters and markers of tissue and liver damage. Spearman correlation analyses correlated clinical and metabolic parameters to coagulation status (**A**–**F**) or tissue damage (**G**–**L**). Volcano plots represent metabolites that have significant (*p* < 0.05) positive (red) or negative (blue) correlations with any of the parameters. Parameters are abbreviated using standard clinical terms.

**Figure 6:**
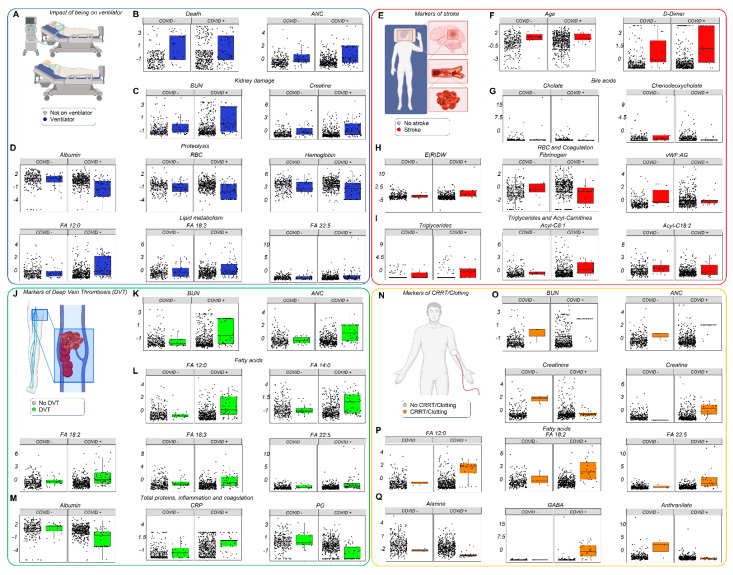
Clinical complications and metabolic/clinical markers. Hospitalized patients, with and without COVID-19, were divided into subgroups depending on clinical complications (e.g., stroke, deep vein thrombosis) and/or interventions (e.g., ventilators, hemodialysis). Mechanical ventilation (**A**–**E**), stroke (**F**–**J**), DVT (**K**–**N**), and hemodialysis (with or without coagulopathy; **O**–**Q** ) in both COVID-19 patients and controls. All metabolites shown in this figure as dot plots are significant by two-way ANOVA (FDR < 0.05).

**Figure 7:**
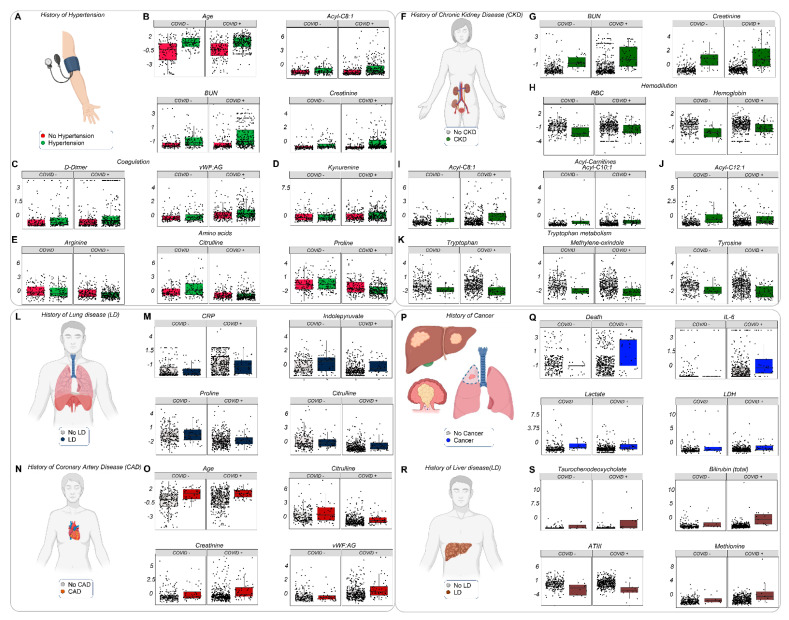
Pre-existing conditions and metabolic/clinical markers. Hospitalized patients, with and without COVID-19, were divided into subgroups depending on clinical history. Specifically, patients were identified who presented with a history of hypertension (**A**–**E**), chronic kidney disease (**F**–**K**), lung disease (**L**–**M**), coronary artery disease (**N**,**O**), cancer (**P**,**Q**), or liver disease (**R**,**S**). All metabolite/clinical variables shown in this figure as dot plots are significant by two-way ANOVA (FDR < 0.05).

**Figure 8:**
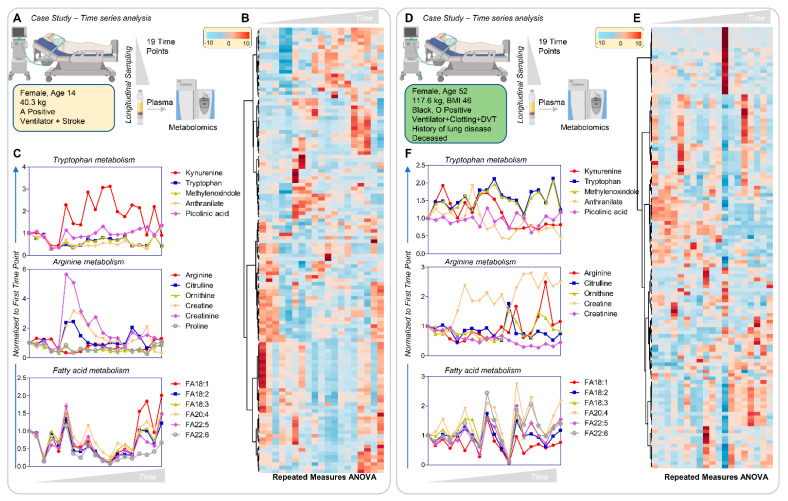
Time-course analysis of two patients with severe COVID-19, one surviving (**A**–**C**) and one dying (**D**–**F**) of disease. Both patients were ventilated with coagulopathic complications, either stroke or deep vein thrombosis. The first patient, a 14-year-old female with no pre-existing conditions, survived at the end of the time course and manifested transient activation of the kynurenine pathway and accumulation of creatinine (kidney dysfunction), which resolved early (**C**). This patient was also characterized by late accumulation of plasma free fatty acids (18C, 20C, and 22C poly- and highly unsaturated fatty acids). The second patient, a 52-year-old female with a history of obesity and lung disease, did not survive COVID-19; no activation of the kynurenine pathway was observed and creatine levels remained elevated.

Overall, these results demonstrate significant up-regulation of creatine metabolism, accompanied by dysregulation of arginine catabolism to proline, polyamines, and citrulline (Figure 1J); also a hallmark of COVID-19-induced endotheliopathy [32]. Interestingly, other markers of endothelial coagulopathy were also significantly increased in COVID-19 patients (Figure 1, Figure 2, Figure 3, Figure 4, Figure 5, Figure 6, Figure 7 and Figure 8), including VWF and its collagen-binding activity (*p* < 0.0001). However, no significant differences in ADAMTS-13 levels or activity were observed; thus, VWF antigen: ADAMTS13 activity ratios were increased (*p* < 0.0001), favoring high-molecular-weight VWF oligomers and increased thrombotic potential. 

### 3.2. Up-Regulation of the Kynurenine Pathway Is Inversely Related to Indole Metabolism

Despite widespread decreases in most amino acids, circulating levels of kynurenine, a tryptophan catabolite (and other kynurenine pathway intermediates) were confirmed [1,4,33,34,35] to be significantly increased in COVID-19 patients as a function of IL-6 levels (Figure 2A,B). In contrast, indole metabolites, which are largely derived from tryptophan metabolism by the gut microbiome, were significantly decreased in COVID-19 patients (Figure 2A). Indeed, plasma levels of tryptophan/indoles and kynurenine were among the top negative and positive correlates with IL-6 levels (Figure 2B, Supplementary Figure S2). IL-6 levels also positively correlated with coagulopathy markers (APTT, D-dimer), and mortality (Figure 2B). Positive correlations with age (Figure 2C) were observed for mortality and hypoxia markers [36], including lactate, purine oxidation products (xanthine, urate), and markers of mitochondrial dysfunction (carboxylic acids citrate, alpha-ketoglutarate, succinate, fumarate), with a role in inflammation and thermogenesis via lipid catabolism [37,38,39]. COVID-19 induced increases in free fatty acids as well as short- and medium-chain acylcarnitine species and decreased long-chain saturated and unsaturated acylcarnitines (Figure 2D,E). Poly- and highly unsaturated fatty acids positively correlated with IL-6 (Figure 2B) and negatively correlated with age (Figure 2C), but correlated positively with markers of kidney dysfunction (BUN), coagulation (vWF levels, plasmin generation—PG rate), and body weight. These results suggest increased lipid mobilization resulting from SARS-CoV-2-induced blood cell membrane vesiculation/lipolysis, as reported [21], and/or adipose tissue lipid catabolism, perhaps as a strategy for assembling viral membranes [6] (Figure 2F). 

Given the role of obesity in COVID-19 outcomes [40], we evaluated metabolomics data in five body mass index (BMI) ranges, from underweight (BMI 13–20) to severely obese (BMI up to 50), highlighting a positive correlation between BMI and several 18, 20, and 22C series mono- and poly-unsaturated fatty acids in COVID-19-positive, as compared to COVID-19-negative patients (significant metabolites shown in Figure 2G, Supplementary Figure S3). Therefore, lipidomics analyses were performed as a function of the lowest (<20) and highest (>38) BMI ranges (n = 15 subjects per group) and results separated by lipid class and fatty acyl-chain composition (Figure 2H–I; Supplementary Table S1). COVID-19 patients, especially those with highest BMI, had significantly higher levels of phosphatidylcholines (PCs), triacylglycerols (TAG), diacylglycerols (DAG), monoacylglycerols (MG), lysophosphatidylethanolamines (LPEs), and phosphatidylserines (PS; Figure 2H); these were particularly enriched in very-long-chain, highly unsaturated fatty acids (20:3, 20:5, 22:5, 22:6) and depleted in 18C series fatty acids (stearic, oleic, linoleic) (Figure 2I).

### 3.3. Effects of Sex, Age, and Ethnicity on the Plasma Metabolome of Hospitalized COVID-19 Patients

Because older male COVID-19 patients have a poorer prognosis, as compared to young females [3,40,41], and given the large size of our cohorts, we tried identifying metabolic and clinical correlates for these variables (Figure 3A–D, Supplementary Figures S4E–J and S5). Aging was associated with increased weight, BMI, kidney dysfunction (creatine, creatinine), and tissue damage (creatine kinase), along with markers of hypercoagulability (VWF:AG, FVIII), fibrinolysis (D-dimer), hyperglycemia, hypoxia and mitochondrial dysfunction (2-hydroxyglutarate, lactate, spermidine, acylcarnitines), purine oxidation (urate), inflammation (CRP), proteolysis (albumin), and anemia (hemoglobin levels, RBC counts), especially in COVID-19 patients (Figure 3A–D). 

Male patients, both with and without COVID-19, had higher RBC counts and hemoglobin levels, lower citrulline and creatine levels, and lower levels of highly unsaturated fatty acids (e.g., eicosapentaenoic, docosapentaenoic, docosahexaenoic acid; Figure 3E–I, Supplementary Figure S5); however, only COVID-positive males, but not females, had increased urate levels (Figure 3J). 

Because RBC count and hemoglobin level were tightly correlated (Supplementary Figure S6) and were affected by both age and sex, we divided both cohorts into sub-groups based on RBC count (Figure 3K); this highlighted a positive correlation between RBC count and kidney damage (BUN, creatinine, guanidinoacetate), total protein level, and glycemia, along with negative correlations with one-carbon metabolites choline and methionine. In these cohorts, race was also associated with inflammation, thromboinflammatory complications, body weight/BMI, and kidney dysfunction; indeed, IL-6, D-dimer, BUN, and creatinine levels were highest in individuals with COVID-19 of African descent (Supplementary Figure S7). In addition, plasma dimethylglycine, indole, and cystine levels were highest in individuals of African descent, whereas kynurenine levels increased in all COVID-19 patients independent of race. Interestingly, ABO blood group status, which is controversially associated with COVID-19 prognosis [42], indicated that the highest kynurenine, GABA, dimethylglycine, and creatinine levels were in blood group O subjects (Supplementary Figure S8). Although our sample size was limited for blood group A COVID-19 patients (n = 111 samples), they had the highest IL-6 levels (Supplementary Figure S8).

### 3.4. Markers of Mortality in Acutely Ill Hospitalized Patients 

While previous studies identified prognostic and disease severity markers in COVID-19 patients, they studied relatively few patients and did not include hospitalized COVID-19-negative patients as controls [1,34,35,43,44,45,46,47,48]. To visualize ranking correlates of mortality, we performed preliminary correlation analyses of both our cohorts (Figure 4A), confirming strong positive correlations between mortality and markers of inflammation, coagulopathy, kidney and tissue damage, and hypoxia. Because death is a non-continuous variable, biomarker analyses were also performed to calculate ROC curves for metabolites and clinical covariates at admission that significantly associated with poor outcomes independent of cohort (Figure 3B–F), or divided into COVID-19 patients and controls (Supplementary Figure S9). Several of the highest-ranking variables (Figure 3) included IL-6, acylcarnitines (especially hexanoylcarnitine), D-dimers, albumin, and tryptophan metabolites.

Because metabolomics data and clinical variables were available for 542 COVID-19 samples, we used 244 randomly selected samples to train a machine learning model to predict mortality in these patients (Figure 4G). Data on training, ROC curves from multivariate models, prediction accuracy, and the top 15 variables fed into the model are shown in Supplementary Figure S10A–B for elaboration with the random forest or SVM algorithm. Overall, the top 10 variables from the random forest algorithm (Figure 4H) showed an AUC of 0.81 (confidence interval 0.71–0.89), resulting in the highest predictive ability with the fewest variables. Using the remaining 298 samples as a test set correctly predicted survival or death of 234 patients, with only five false positives (i.e., predicted to die, but survived) and 59 false negatives (i.e., predicted to survive, but died), demonstrating a 78% accuracy of the model, with high specificity (>95%), but moderate sensitivity (<70%).

### 3.5. Metabolic and Clinical Correlates to Markers of Coagulopathy and Tissue Damage

Correlation analyses (Spearman) identified clinical and metabolic correlates to coagulation parameters, including D-dimer, APTT, INR (>96% positive correlation with APTT, thus excluded from the volcano plot), FVIII, TG, VWF:AG, and VWF:collagen-binding activity (Figure 5A–F). IL-6, age, metabolites linked to oxidant stress and sulfur metabolism (cystine), acylcarnitines (markers of mitochondrial dysfunction [49] and platelet activation [50,51]), and tryptophan and its metabolites, were top correlates to the coagulation parameters (Figure 5A–F). 

Similarly, correlating metabolites and clinical parameters to markers of tissue damage (CK, LDH), inflammation (CRP), liver damage (ALT, AST), and proteolysis/hemodilution (albumin) identified a strong interaction with arginine/proline metabolism, ferritin/hemoglobin/RBC counts, and tryptophan/kynurenine metabolism (Figure 5G–L). Interestingly, conjugated bile acids, well-established markers of liver inflammation [52], were positively correlated with liver transaminases (Figure 5J–K).

### 3.6. Clinical and Metabolic Correlates to Clinical Complications: Ventilators, Stroke, Deep Vein Thrombosis (DVT), and Hemodialysis

Leveraging the manually curated clinical records for the enrolled patients, we identified clinical and metabolic markers correlating with mechanical ventilation (Figure 6A–E; Supplementary Figure S11), stroke (Figure 6F–J), DVT (Figure 6K–N), and hemodialysis (with or without coagulopathy; Figure 6O–Q and Supplementary Figure S12) in both COVID-19 patients and controls. In all cases, the top markers were related to kidney dysfunction (BUN, creatinine), proteolysis/hemodilution (albumin, RBC count, hemoglobin, fibrinogen), free fatty acids (dodecanoic, linoleic, linolenic, docosapentaenoic), acylcarnitines, triglycerides, and amino acid metabolism (especially tryptophan, choline, and GABA). Trends observed in controls were more dramatic in COVID-19 patients presenting with similar manifestations.

### 3.7. The Effects of Clinical History and Pre-Existing Conditions on the Metabolome and Clinical Phenotype of Acutely Ill Hospitalized Patients 

Pre-existing conditions, including obesity, cardiovascular disease, kidney disease, cancer, and diabetes, are all associated with poorer prognosis in COVID-19 [53]. Meta-analysis of our cohorts (Figure 7A–S, Supplementary Figure S12) indicated that older subjects are more likely to present with a history of hypertension, coronary artery disease, and/or diabetes (Figure 7A,O; Supplementary Figure S12A,B). Hypertension, chronic kidney disease, lung disease, and coronary artery disease share altered tryptophan and arginine/proline/citrulline metabolism, trends exacerbated by COVID-19. Carnitine metabolism and aromatic amino acids were increased in patients with a history of kidney disease (Figure 7F–K), whereas cancer was accompanied by increased lactate (perhaps resulting from a Warburg phenotype; Figure 7Q). A history of liver disease was accompanied by increased levels of conjugated bile acids (e.g., taurochenodeoxycholate), total bilirubin, and methionine (Figure 7S). Finally, a history of diabetes was associated with increased lactate and lactoyl-glutathione levels, the latter a marker of glyoxylase damage (Supplementary Figure S13).

### 3.8. Longitudinal Sampling in Severe COVID-19 Patients

Sampling at admission allowed us to collect longitudinal samples from some patients. The results from three severe COVID-19 cases, only two of whom recovered, are presented here. Figure 8 (vectorial version in Supplementary Figures S14–S16, data in Supplementary Table S1) shows hierarchical clustering of metabolites as a function of time (19 time points for two patients and 21 for the third patient). These three patients were female, 14, 45, and 52 years old, of different ethnicity and BMI. Despite similar disease severity (e.g., all mechanically ventilated, with either stroke, clotting, or DVT manifestations), only the surviving patients manifested a spike in kynurenine levels throughout their course, which was not observed in the patient who died (Figure 8C,F). Increased creatine/creatinine eventually resolved in the surviving patients, but not in the patient who died. The surviving patients also manifested increased free fatty acid levels at the latest time points, especially poly and highly unsaturated fatty acids of the 18, 20, and 22C series; in contrast, the non-surviving patient exhibited late accumulation of acylcarnitines and amino acids which did not resolve (Supplementary Figure S16). 

## 4. Discussion

The present study provides the most extensive metabolomics analysis of COVID-19 patients to date, including 831 samples at admission from hospitalized patients with and without COVID-19 and 59 longitudinal samples from three case studies. Previous metabolomics studies on COVID-19 were not powered to characterize the effects of other variables critical for disease severity and prognosis. As examples, evaluations of biological (e.g., sex [54], age [55], ethnicity [56], body mass index [57], blood group [42]) and clinical (e.g., obesity, diabetes, cardiovascular disease, kidney disease) [53] characteristics are necessary to define independent and overlapping metabolic findings in COVID-19 and other acute diseases. To this end, in some cases, we performed sub-analyses focusing on one variable at a time, such as sex [3,41] or inflammation (e.g., circulating interleukin-6 (IL-6) levels) [1,4]. 

Leveraging the combination of large omics datasets from COVID-19 patients and hospitalized (non-healthy) controls with manually curated clinical records, novel metabolic correlates to biological variables and patient characteristics were identified in this study; these results confirm and significantly enhance previous efforts in this disease [58,59]. For example, despite a positive correlation with weight and BMI, aging was accompanied by decreased circulating levels of several poly- and highly unsaturated fatty acids (PUFAs), consistent with reported age-dependent declines in unsaturated fatty acids in healthy blood donors [23] and fatty acid desaturase activity, with functional implication in hematopoiesis [60] as well as inflammatory and immune modulation [61]. PUFAs and their bioactive derivatives (e.g., hydroxyoctadecadienoic and hydroxyeicosatetraenoic acid) have been identified as modulators of inflammation as well as acute and chronic immune response [61]. Dysregulated production of these immune-regulating lipid mediators (eicosanoids and related docosanoids) was observed in patients with influenza [62] and COVID-19 [63]. Decreased plasma PUFA levels in older and obese patients could contribute to their poor prognosis after SARS-CoV-2 infection, potentially benefiting from therapeutic treatment with intravenous omega-3 PUFAs to normalize PUFA levels, thereby increasing the production of anti-inflammatory and pro-resolving immune lipid modulators [64]. 

Aging was also accompanied by increased markers of hypoxia (e.g., lactate, citrate, alpha-ketoglutarate, fumarate), indicative of progressive mitochondrial dysfunction [65]. Given the role of these metabolites in immunometabolism [37], older patients also demonstrated increased inflammation, especially COVID-19 patients, accompanied by poorer outcomes. Similarly, purine catabolism and oxidation products (e.g., urate and xanthine), hallmarks of ischemic [39] and hemorrhagic [36] hypoxic organ damage, increased with age. Importantly, mitochondrial activity, aging, and inflammation are all associated with hypercoagulabiity [49], harmonizing our observational results with the known increased incidence of thromboembolic complications in COVID-19.

In contrast, aging, especially in COVID-19 patients, was accompanied by altered levels of free fatty acids and acylcarnitines. The former may fuel viral membrane synthesis through increased ATP production, which may be sustained by lipid mobilization from adipose tissue and other sources, similar to observations in trauma patients [66] and following the pathological vesiculation of RBC membranes [21]. Because obesity also leads to poor outcomes in COVID-19, lipidomics analyses of 60 subjects with the highest and lowest BMIs allowed identification of obesity-related lipid signatures in COVID-19 patients. In particular, neutral lipids (MG, DAG, TAG) and phospholipids (PC and LPE) were mobilized; the latter may result from the release of methyl-groups from LPCs to meet one carbon demands for viral nucleotide synthesis or repair of oxidant-induced isoaspartyl damage [67]. This hypothesis is supported by the observed increase in plasma methionine in the context of lower levels of most other amino acids. Similar to other studies, low levels of most amino acids were observed

These metabolic observations of aging and obesity were exacerbated in COVID-19 patients and were consistent with disease severity, as indicated by clinical records and clinical measurements of markers of inflammation (IL-6, CRP), coagulopathy (D-dimers, APTT, INR, FVIII, VWF:AG, VWF:collagen-binding activity, VWF:ADAMTS-13 activity ratios, thrombin and plasmin generation), and renal dysfunction (BUN, creatinine). Metabolic correlates of these clinical parameters are provided in this study, as part of the efforts aimed at compiling an encyclopedic characterization of metabolism in health and disease. For example, we found strong negative correlations between kidney dysfunction and circulating amino acid levels, as possible indicators of decreased renal reabsorption [29,30,68] and hemodilution. As another example, positive correlations between pro-inflammatory conjugated bile acids and liver transaminases support prior findings of mechanistic interactions of these metabolites with IL-1beta and hepatic stress [52]. Interestingly, these metabolites were also associated with coagulopathy in trauma/hemorrhagic shock [69], and with microbiome dysbiosis related to iron metabolism [70], observations informing the correlations in our study between ferritin levels, acute-phase response proteins (CRP), and conjugated bile acids. 

Besides aging and inflammation, other factors are also associated with poor outcomes in COVID-19. For example, the expression levels of angiotensin-converting enzyme 2 (ACE2) receptor in enterocytes modulate disease severity, in that viral entry into cells is mediated by pairing of ACE2 with the viral spike protein [71]. Notably, we confirm that arginine/proline/citrulline metabolism is an important pathway affected by COVID-19 [1,4,72], which not only depends on kidney function, but also on enterocytes [73]. Low plasma arginine and arginine bioavailability were observed in children and adults with COVID-19 [72]. These patients had reduced T-cell proliferative capacity in vitro that was partially, but significantly improved with arginine supplementation [74]. Furthermore, arginase to nitric oxide (NO) synthase activity may influence the pro-/anti-inflammatory state of gut resident macrophages [75]. In addition, circulating levels of arginine pathway metabolites can be affected by RBC arginase activity [21], which is in turn affected by oxidant stress and aging [76] and can contribute to COVID-19-induced endotheliopathy [32]. In the current study, arginine was significantly lower in COVID-19-positive patients compared to COVID-19-negative critically ill hospitalized patients. Taken together, low plasma arginine in COVID-19 patients may contribute to endothelial dysfunction via decreased NO generation as well as alter immune response, which could potentially be improved with arginine supplementation. 

Endothelial dysfunction is a common finding in patient populations most at risk for severe disease (i.e., obesity, diabetes, hypertension). The endothelium is directly affected by SARS-CoV-2 infection, resulting in damage to the vasculature [77,78]. The endothelium can also be indirectly affected in COVID-19 through the overstimulation of the immune system, resulting in cytokine storm, endothelial activation, and capillary leak. The damaged and overstimulated endothelium results in a shift in the vascular equilibrium towards more vasoconstriction with subsequent organ ischemia and circulatory collapse along with upregulation of the coagulation cascade promoting a pro-thrombotic state, which is likely exacerbated in patients with pre-existing endothelial dysfunction [79,80]. 

Indole metabolites of microbial origin [81] were also significantly decreased in COVID-19 patients, especially in those with the poorest outcomes. These decreases may result from tryptophan depletion as a function of kynurenine pathway activation in COVID-19 [1,33,34,41], especially in older males. We confirmed that kynurenine levels correlated with SARS-CoV-2 infection, disease severity, and mortality. Indeed, IL-6 levels and kynurenine/tryptophan ratios were among the top predictors of mortality in COVID-19 patients, confirming previous targeted analyses [35] of our larger, independent cohort. However, as activation of interferon responses appear necessary for eliciting adaptive immunity against COVID-19 [46], it is interesting that, in our longitudinal blood collections of the COVID-19 patients who died, plasma kynurenine levels did not increase. In contrast, because some metabolites in the kynurenine pathway are neurotoxic (e.g., picolinic acid, quinolinic acid) [82], uncontrolled activation of this pathway may contribute to some neurological comorbidities of COVID-19 (e.g., brain fog, weakness, fatigue). Interestingly, declines in tryptophan-derived *de novo* nicotinamide synthesis are associated with aging and inflammation [83], suggesting that nutritionally replenishing NAD reservoirs (e.g., nicotinamide riboside) may be therapeutic in facilitating recovery from severe COVID-19 [84]. 

Depleting tryptophan to promote kynurenine synthesis may also lead to serotonin depletion, a key component of platelet-dense granules with a role in platelet activation [85]. This is relevant given the importance of coagulopathy in COVID-19, with increased plasma levels of FVIII, D-dimers, and VWF (i.e., increased VWF:collagen-binding activity, increased VWF:ADAMTS-13 activity ratio), which are among the top correlates of mortality in our cohort. In addition, inflammation negatively correlated with albumin levels, perhaps due to inflammation-induced proteolysis, agreeing with previous reports that albumin predicts all-cause and cardiovascular mortality in chronic kidney disease patients [86]. Albumin strongly correlated with total protein and hemoglobin levels, which were also among the top correlates with kidney dysfunction, thereby strengthening the evidence supporting RBC contributions to kidney physiology [31]. In contrast, no major effects of ABO blood group were noted in our cohort, except for a link to IL-6 levels (highest in blood group A, corroborating prior evidence relating to increased disease severity [42]). Not surprisingly, ABO blood group was also linked to patient ethnicity in our cohort, which correlated with increased inflammation (IL-6), D-dimers, creatinine, and cystine (oxidant stress) in individuals of African descent. 

Altered lipid metabolism is a common finding in patients with COVID-19 [59,87]. Activation of phospholipase A2 increases the formation of lysophospholipase A2 liberating free fatty acids, particularly PUFAs, while producing lysophospholipids, which are necessary for viral replication [88]. Plasma lipididomic profiles reflect all secreted and non-secreted lipid sources found in systemic circulation. Sphingosine-1-phosphosphate (S-1-P), a bioactive lipid with anti-inflammatory actions on endothelial cells [89], was decreased in our previous study comparing COVID-19 patients to healthy controls [1] and subsequently identified as a negative biomarker of disease severity in COVID-19 patients [90]. This observation was confirmed in the current study, supporting the hypothesis that normalizing S-1-P could improve vascular function and disease symptomology.

A recent study comparing healthy controls to COVID-19 patients suggest increased secretion of monosialodihexosylganglioside (GM3)-enriched exosomes (increased levels of sphinomyelins (SMs) and reduced DAGs), altering CD4+ T-cell activation, resulting in immunosuppression [59]. In the current study, SM was decreased and DAG was increased. Differences may be attributable to the health status of the reference group (healthy controls vs. acutely ill hospitalized patients). Additionally, a study of 103 symptomatic COVID-19 subjects from northern Italy were compared to 32 and 26 non-COVID-19 patients with (sick control) and without symptoms (healthy control). The main finding was altered lipid metabolism with similar increases in fatty acid and triglyceride concentration along with decreased sphingomyelin; however, the authors observed decreased DAG and phospholipids (PC, PE, and PI) and a larger increase in lysophospholipid concentration. [87] The observed differences could be due to different study populations or sample analysis. 

Finally, as a proof-of-principle, we entered admission data (clinical and metabolomics) into machine learning algorithms, randomly selecting approximately half of the COVID-19 patient cohort as a training set and the other half as a test set. The resulting model exhibited high specificity (>95%), but moderate sensitivity (~70%). The prediction accuracy of these models may be affected by clinical contributors to the metabolic heterogeneity of hospitalized patients, such as elements of their medical history. Nonetheless, we report here for the first time that metabolic phenotypes of COVID-19 patients were most extreme in patients presenting with a history of hypertension, chronic kidney disease, cancer, coronary artery disease, or lung disease. Future research integrating metabolomics with microbiomics datasets could enhance our understanding on how the gut flora contributes to the pathology and disease severity in patients infected with SARS-CoV-2.

There are several limitations due to the inherent nature of the study design. Metabolomic and lipidomic analysis was performed on leftover, discarded plasma used for PT analysis. Although extreme care was taken to freeze the residual sample as rapidly as possible, there was variability in the amount of time the specimen was stored at 4 C. Additionally, the time of day as well as fasting status was not standardized, which could increase variability in the observed metabolite concentrations [91,92,93]. Because both the control (SARS-CoV-2-negative) and the SARS-CoV-2-positive subjects were critically ill, hospitalized patients, it is unlikely that any of the aforementioned variables could explain the observed metabolic differences described in this manuscript.

Taken together, the extensively detailed information in this large, prospective, observational study will support future mechanistic studies and data sharing opportunities to enhance understanding of the plasma metabolism in COVID-19 and other acute critical illnesses.

## Data Availability

All raw data are available and provided in the supplemental table and raw data are available upon reasonable request to the corresponding author.

## Supplementary Materials

The following are available online at https://www.mdpi.com/article/10.3390/cells10092293/s1, Supplementary Figure.pdf: including Figures S1–S16, Table S1: Metabolomics data.
