# Supplementary material for: Biological and Clinical Factors Contributing to the Metabolic Heterogeneity of Hospitalized Patients with and without COVID-19
Source: Cells. 2021 Sep 2;10(9):2293. doi: 10.3390/cells10092293 (PMC8467961; doi:10.3390/cells10092293)
Supplement: Supplementary file 1 [file cells-10-02293-s001.zip › Supplementary Figures.pdf]

SUPPLEMENTARY MATERIAL

TABLE OF CONTENTS

SUPPLEMENTARY FIGURES..2

SUPPLEMENTARY FIGURE S1..2
SUPPLEMENTARY FIGURE S2..3
SUPPLEMENTARY FIGURE S3..5
SUPPLEMENTARY FIGURE S4..6
SUPPLEMENTARY FIGURE S5..8
SUPPLEMENTARY FIGURE S6..9
SUPPLEMENTARY FIGURE S7..11
SUPPLEMENTARY FIGURE S8..12
SUPPLEMENTARY FIGURE S9..13
SUPPLEMENTARY FIGURE S10..14
SUPPLEMENTARY FIGURE S11..15
SUPPLEMENTARY FIGURE S12..16
SUPPLEMENTARY FIGURE S13..17
SUPPLEMENTARY FIGURE S14..18
SUPPLEMENTARY FIGURE S15..19
SUPPLEMENTARY FIGURE S16..20

SUPPLEMENTARY DATA TABLE *(PLEAE REFER TO "LEGEND" SHEET WITHIN THE FILE)*..................**XLSX**

SUPPLEMENTARY FIGURES

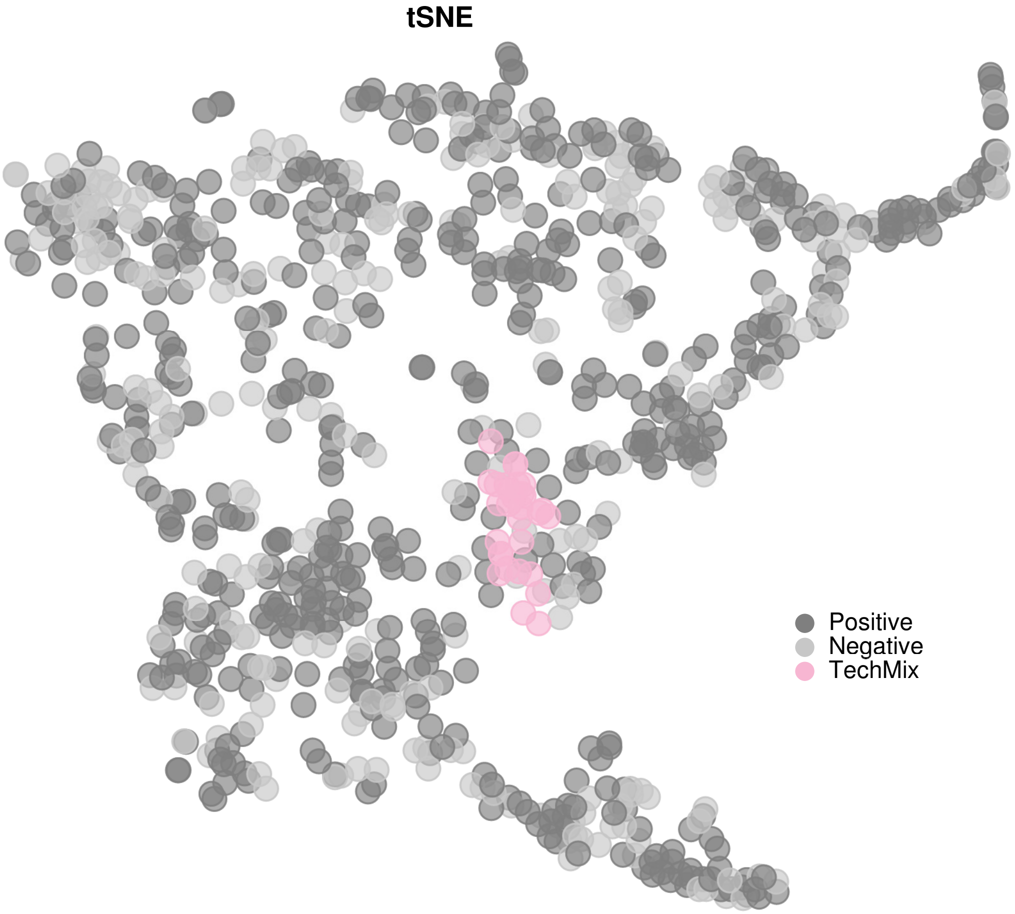

Supplementary Figure S1 – tSNE Analysis of Metabolomics data confirms quality of the analysis, with technical mixes clustering together (pink).

Impact of IL-6

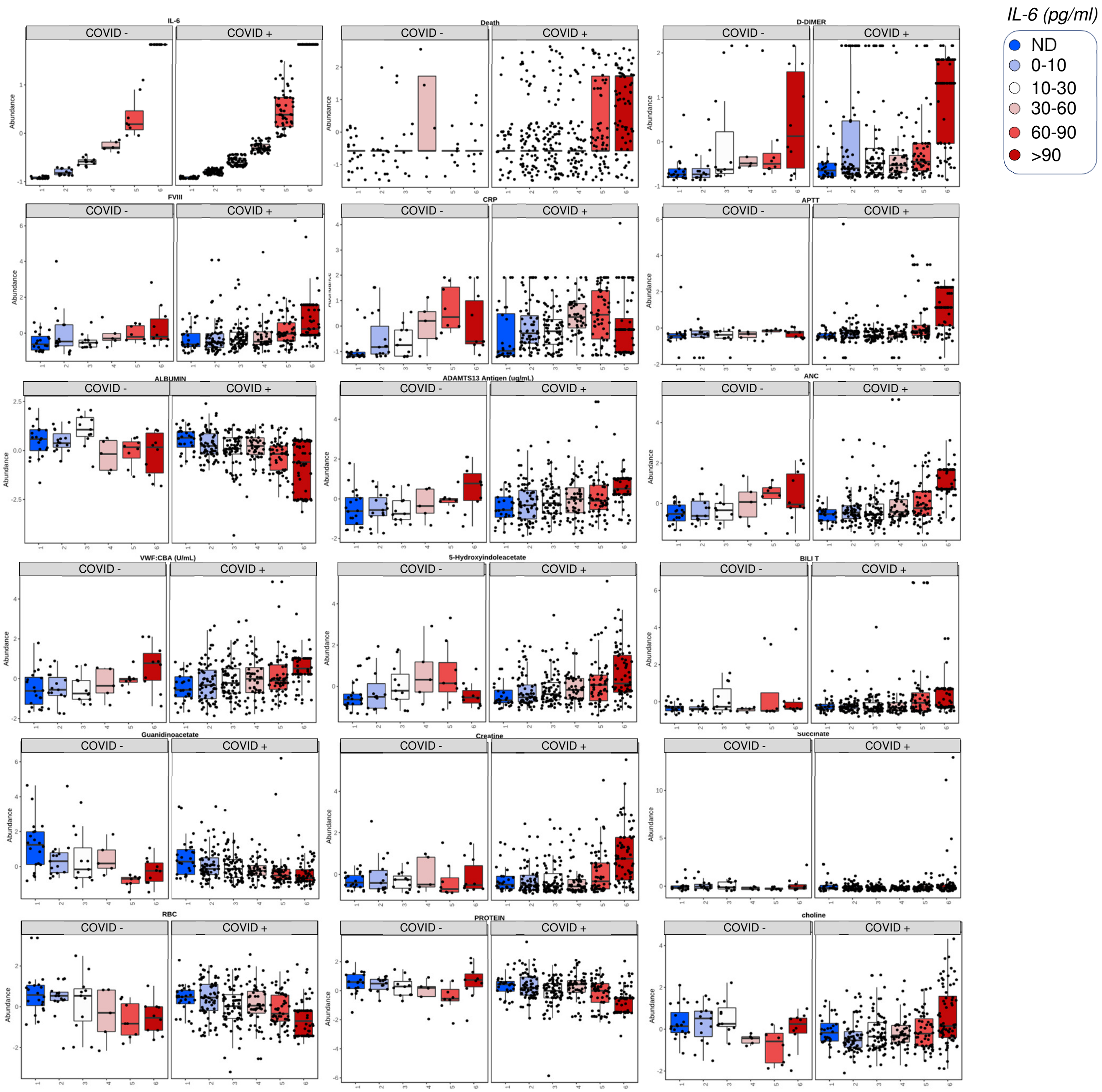

continues →

Impact of IL-6

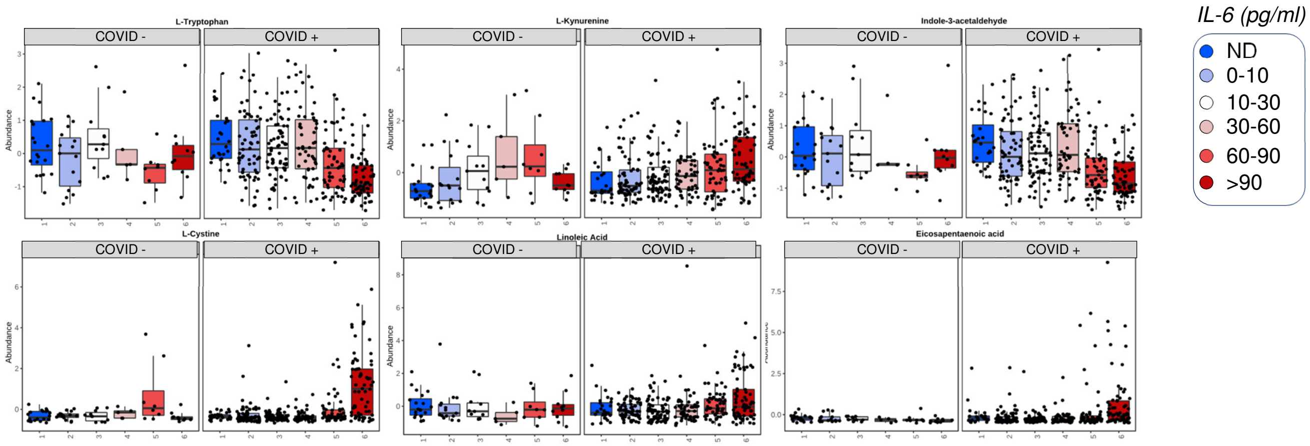

Supplementary Figure S2 – Markers of blood group in COVID negative (-) and positive (+) patients, as determined by Two-way ANOVA.

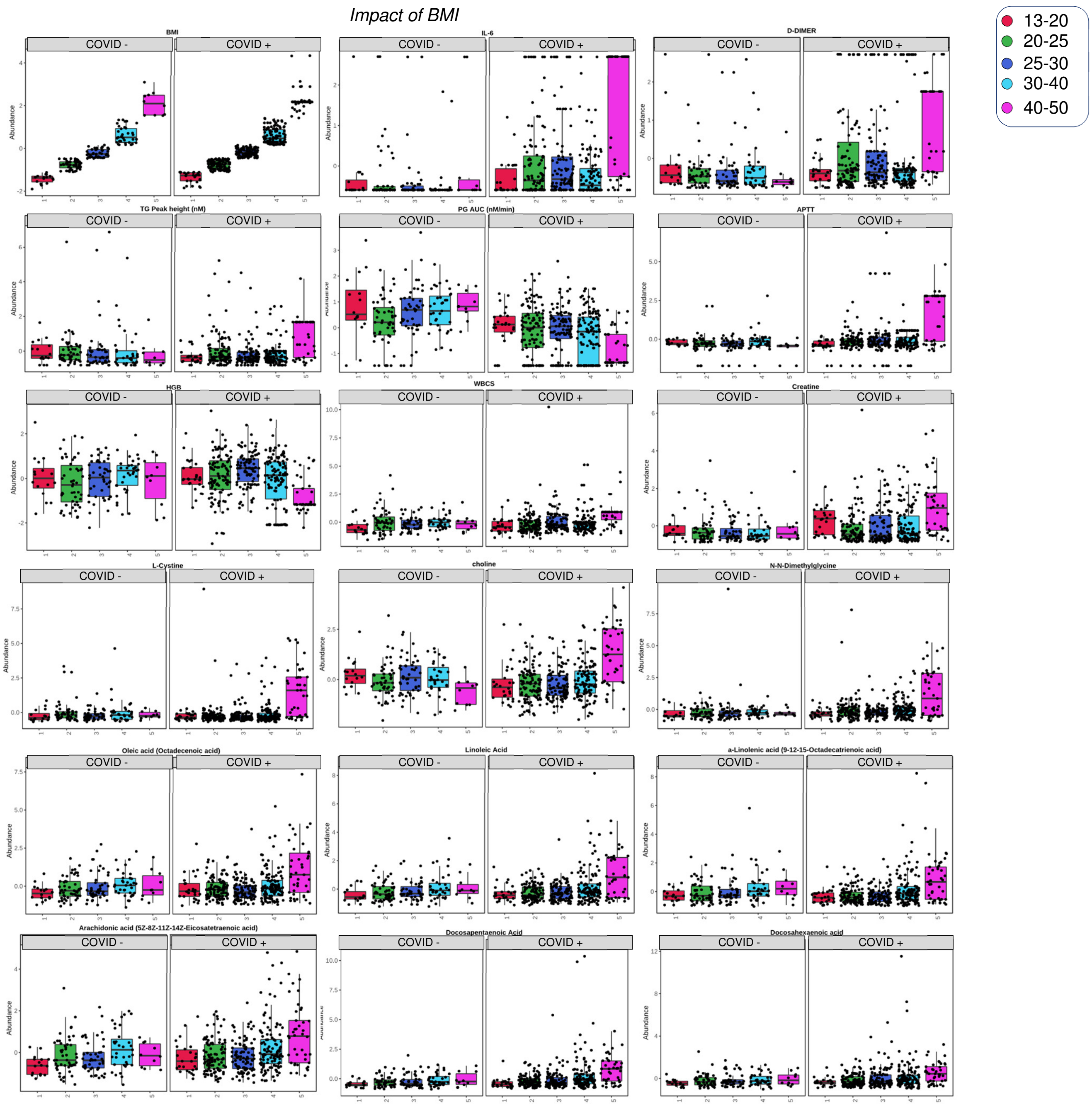

Supplementary Figure S3 – Markers of BMI in COVID negative (-) and positive (+) patients, as determined by Two-way ANOVA.

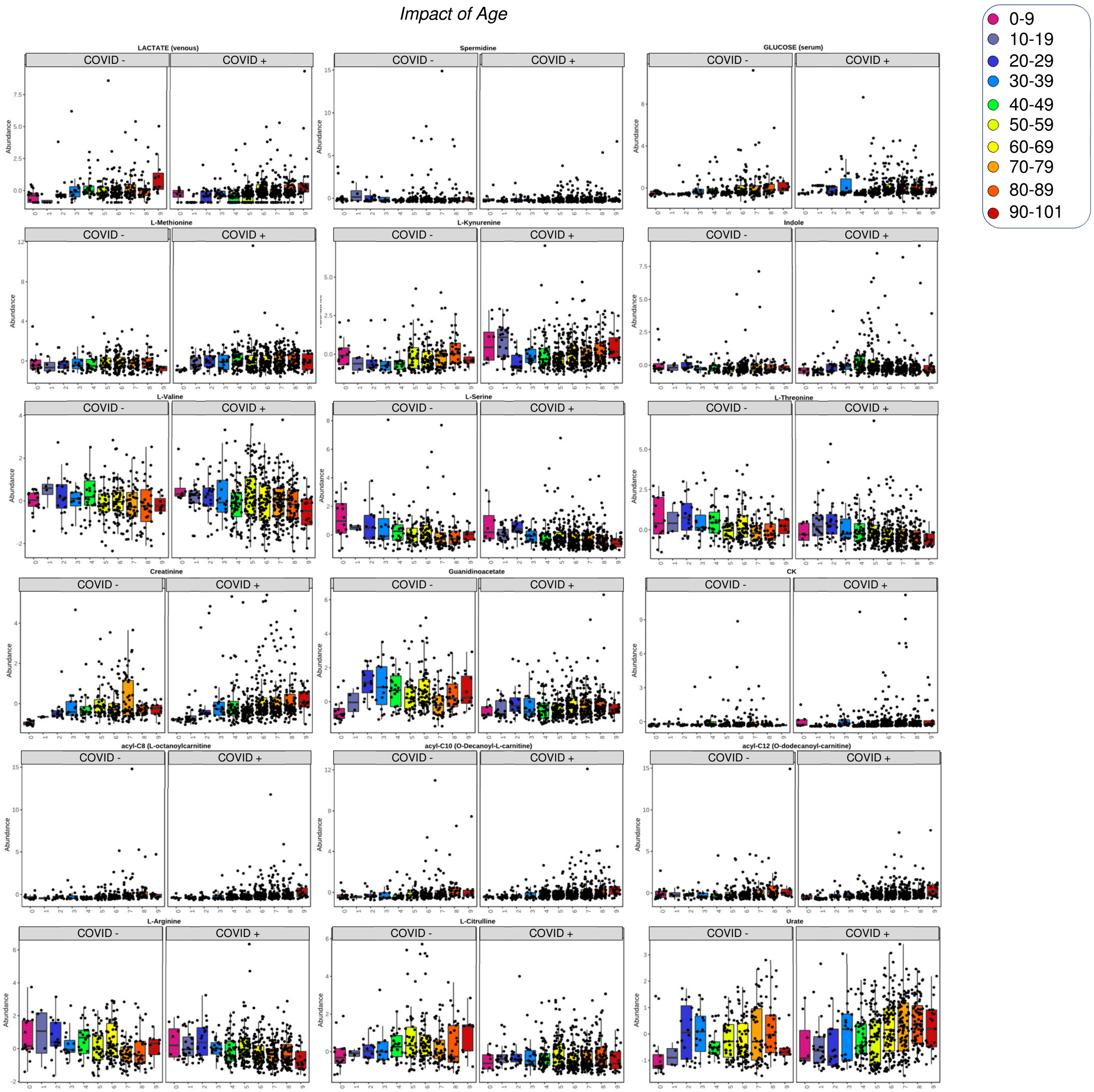
Impact of Age
0-9
10-19
20-29
30-39
40-49
50-59
60-69
70-79
80-89
90-101
LACTATE (venous)
Spermidine
GLUCOSE (serum)
L-Methionine
L-Kynurenine
Indole
L-Valine
L-Serine
L-Threonine
Creatinine
Guanidinoacetate
CK
acyl-C8 (L-octanoylcarnitine
acyl-C10 (O-Decanoyl-L-carnitine)
acyl-C12 (O-dodecanoyl-carnitine)
L-Arginine
L-Citrulline
Urate
COVID -
COVID +
Abundance

continues →

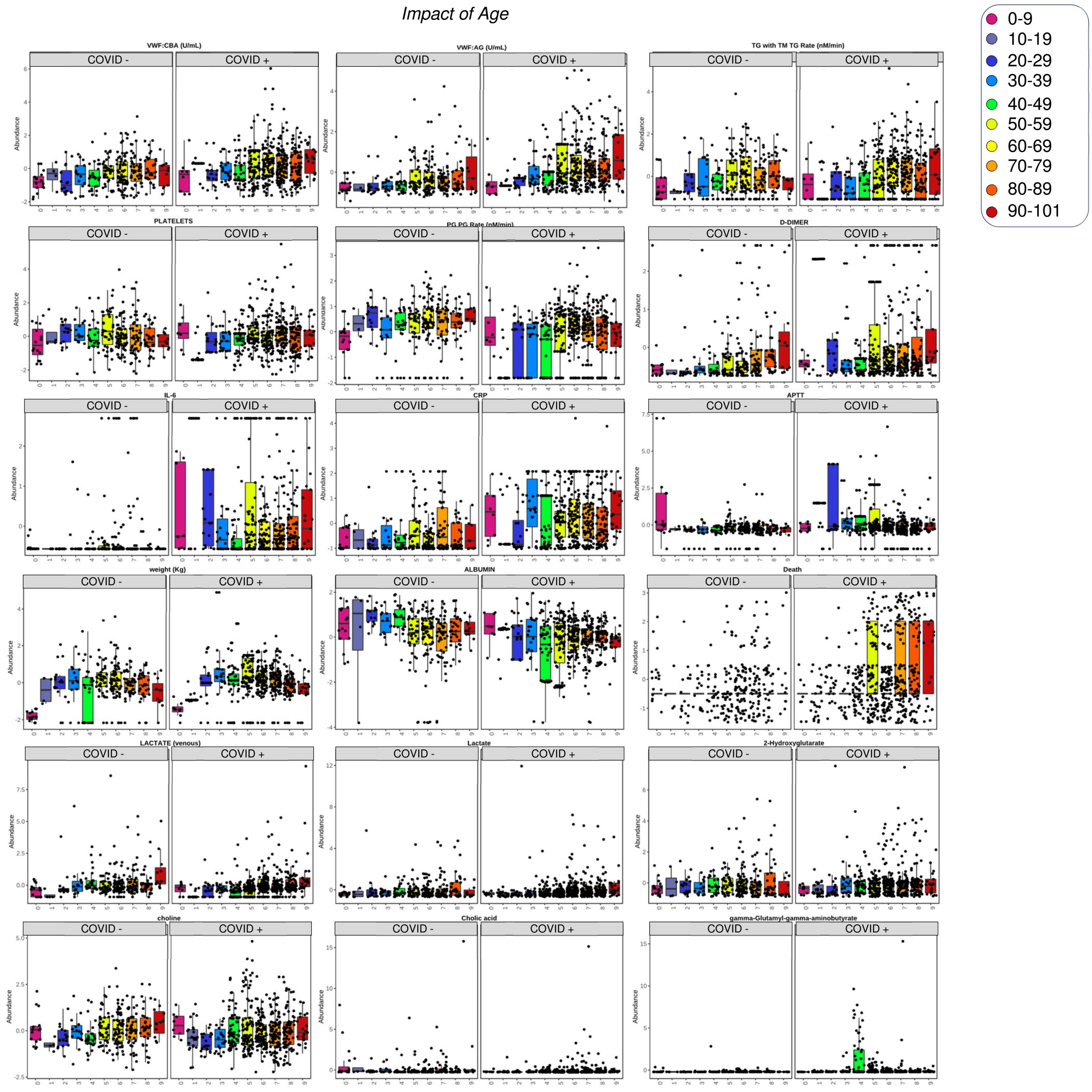

Supplementary Figure S4 – Markers of age in COVID negative (-) and positive (+) patients, as determined by Two-way ANOVA.

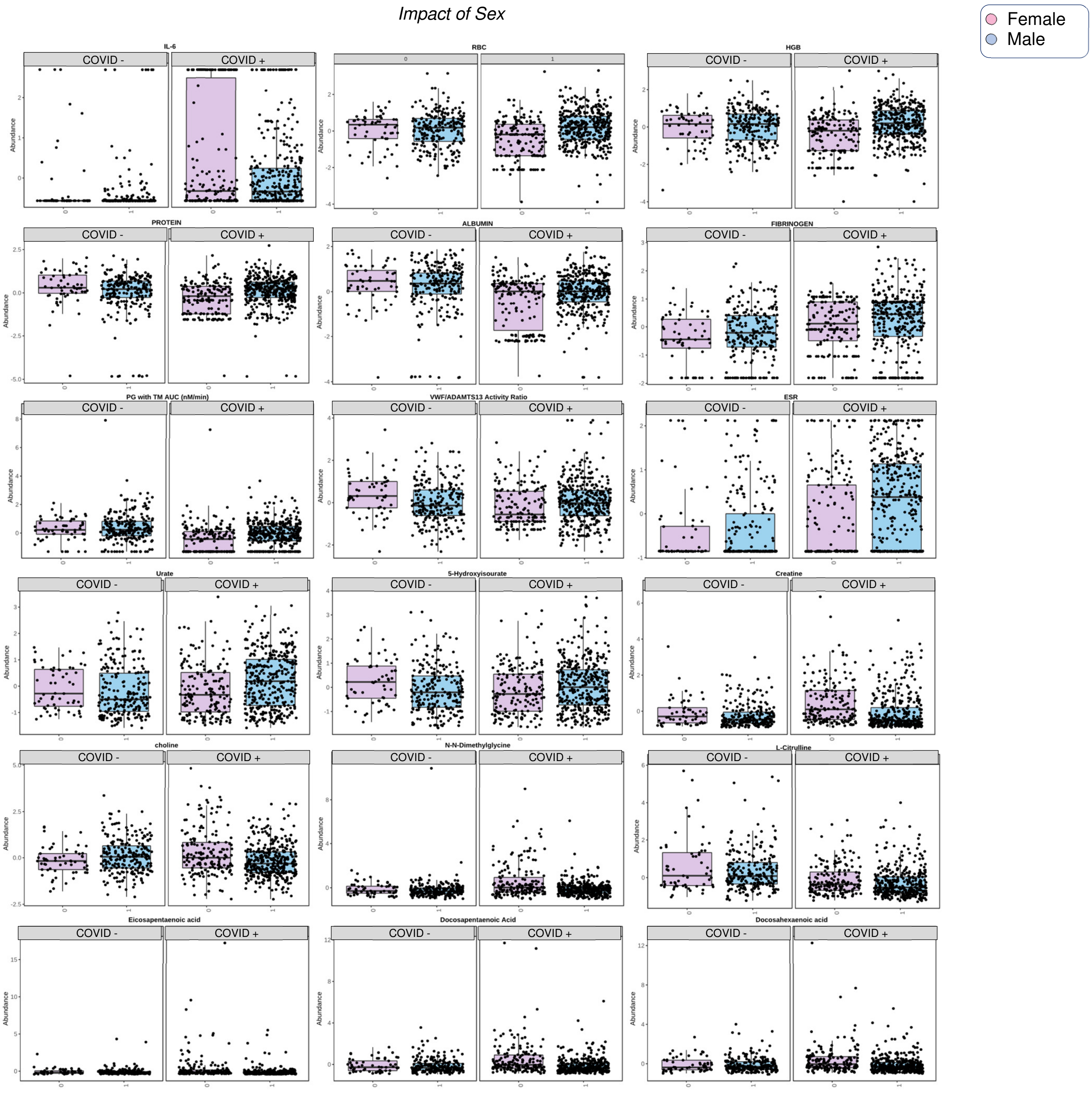

Supplementary Figure S5 – Markers of sex in COVID negative (-) and positive (+) patients, as determined by Two-way ANOVA (0 = female; 1 = male).

Impact of RBC counts (1x 10^6/µl)

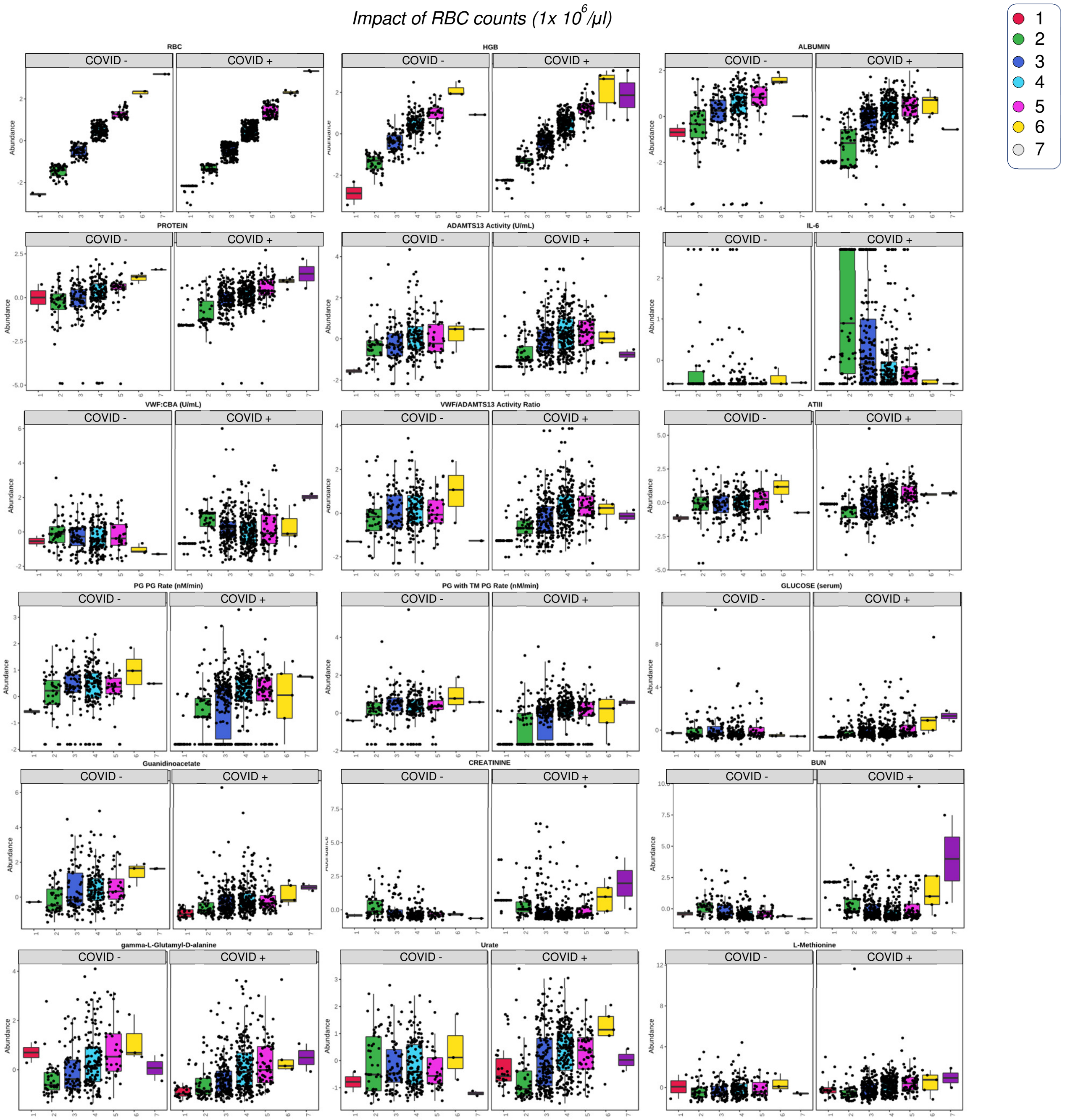

continues →

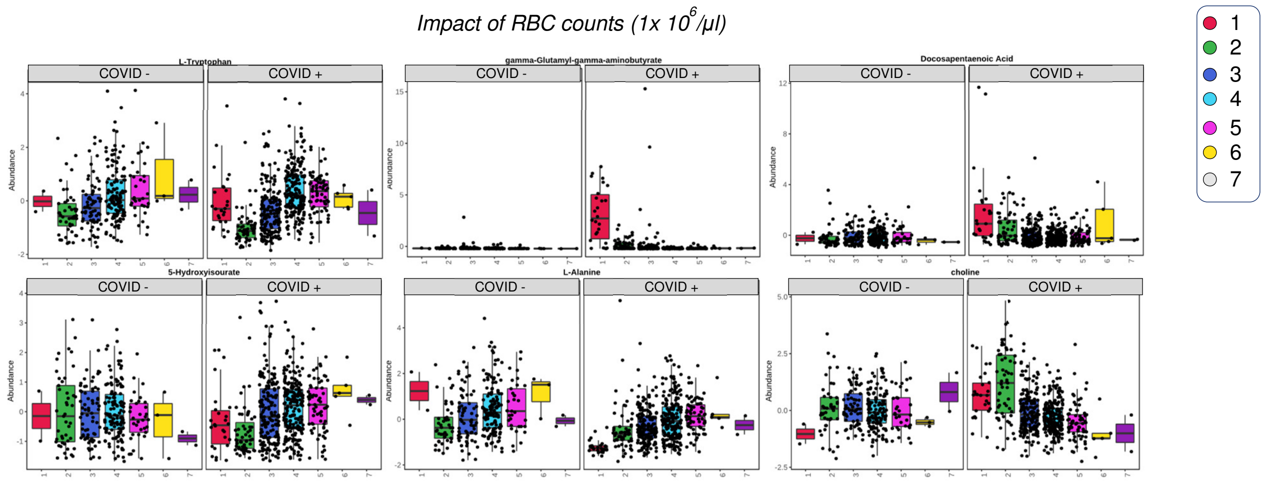

Supplementary Figure S6 – Markers of RBC counts in COVID negative (-) and positive (+) patients, as determined by Two-way ANOVA (numbers in the legend indicate RBC count in millions per microliter).

Impact of Race

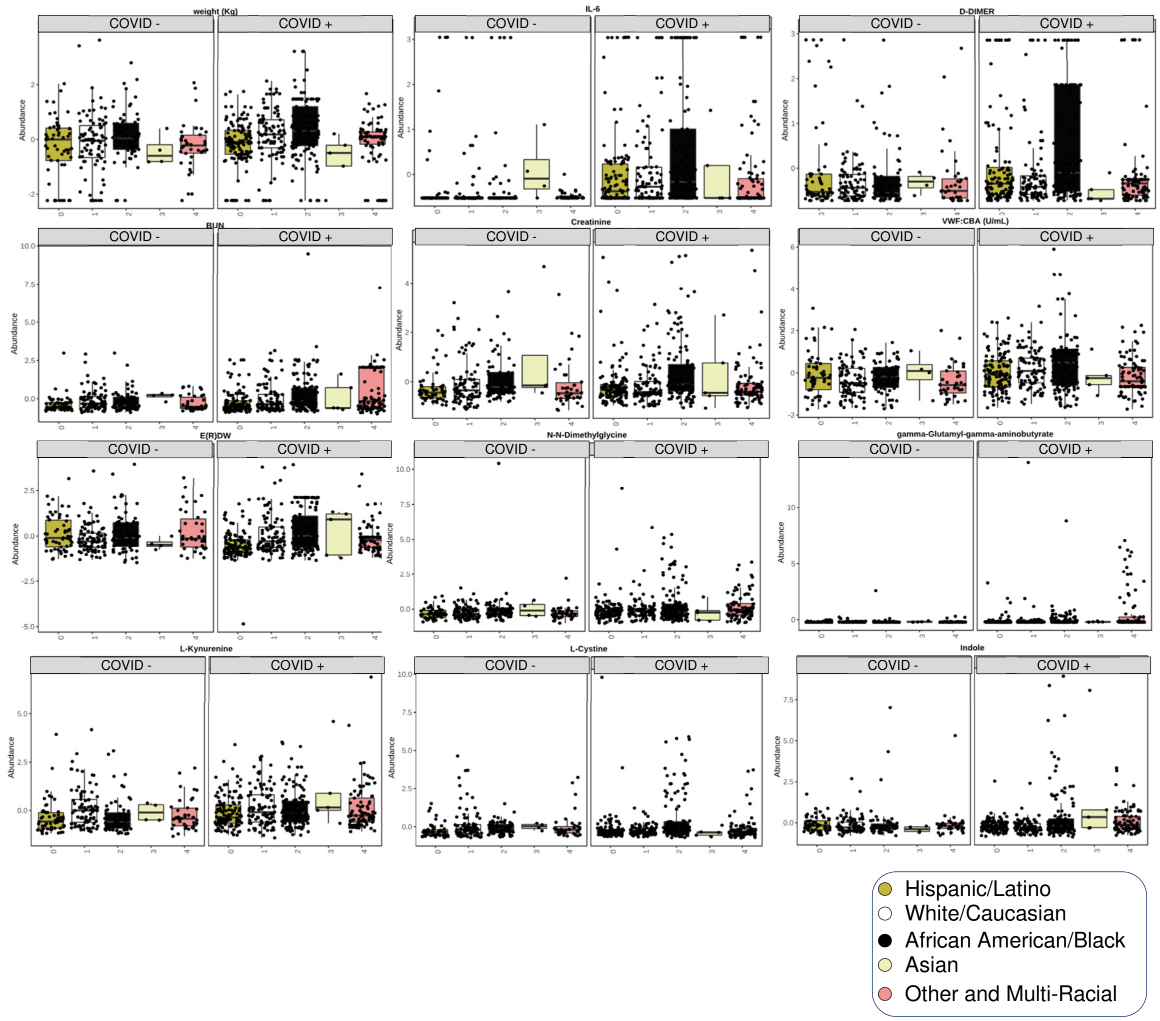

Supplementary Figure S7 – Markers of race in COVID negative (-) and positive (+) patients, as determined by Two-way ANOVA.

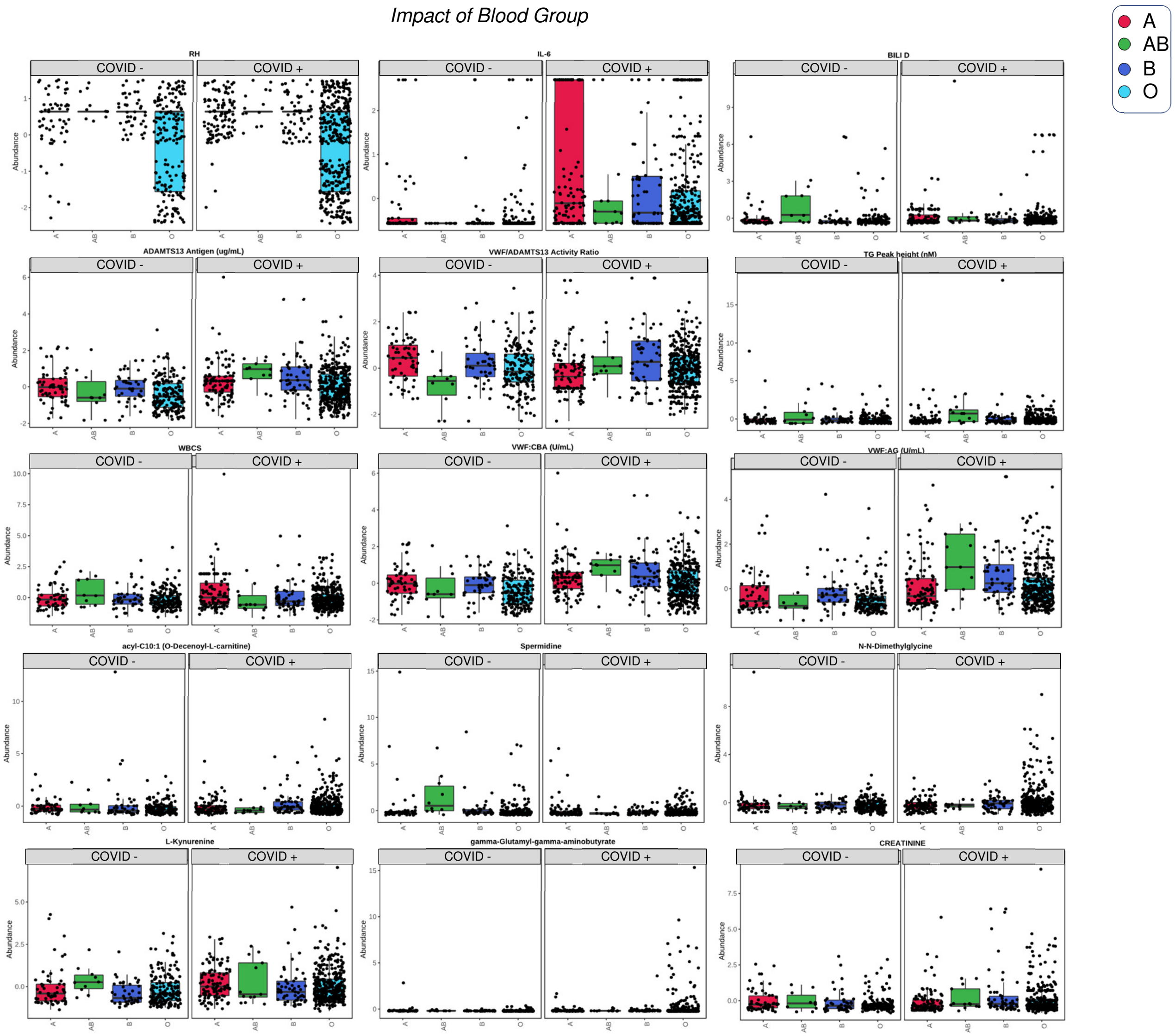

Supplementary Figure S8 – Markers of blood group in COVID negative (-) and positive (+) patients, as determined by Two-way ANOVA.

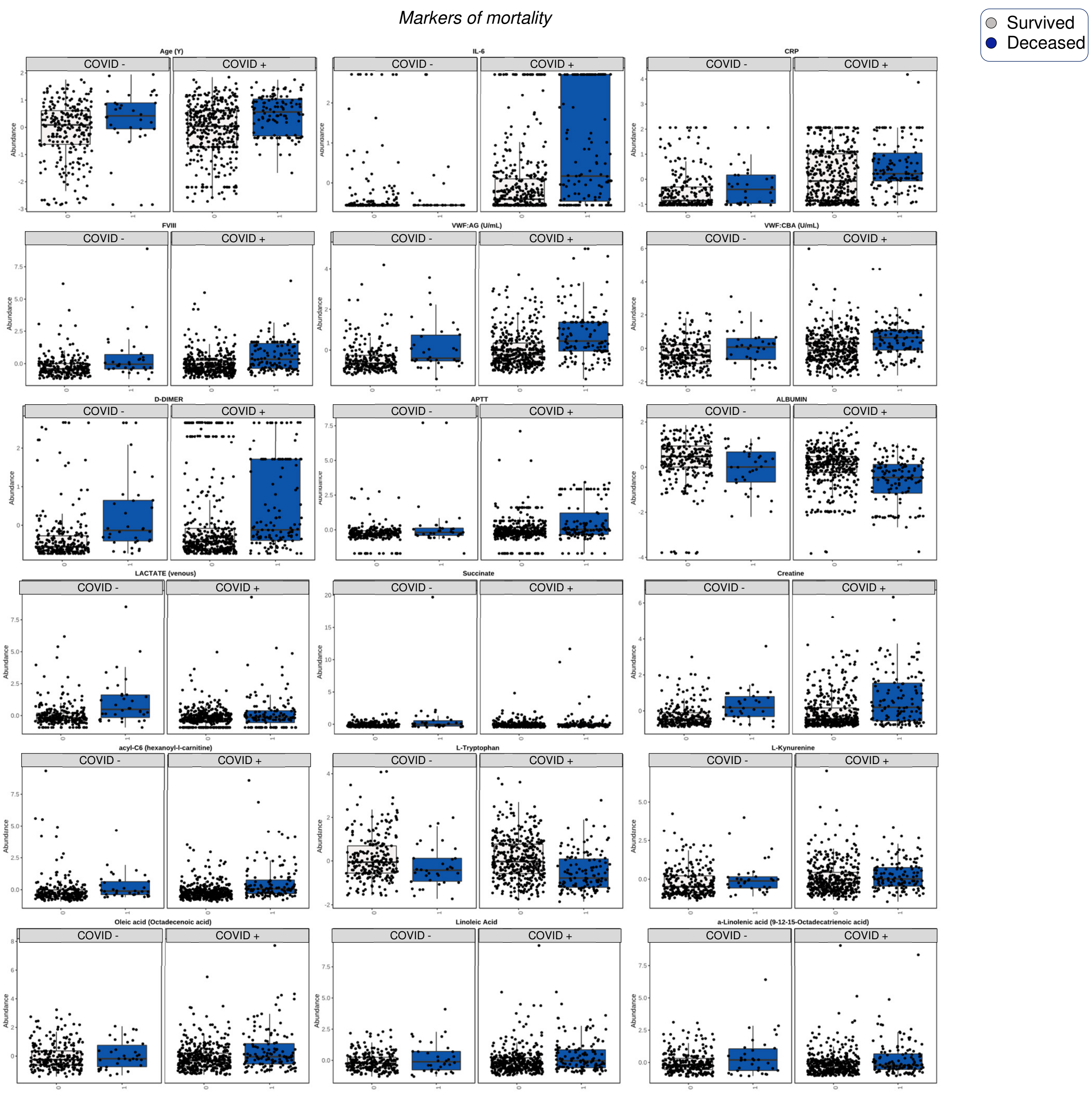

Supplementary Figure S9 – Markers of mortality in COVID negative (-) and positive (+) patients, as determined by Two-way ANOVA (0 = survived; 1 = dead).

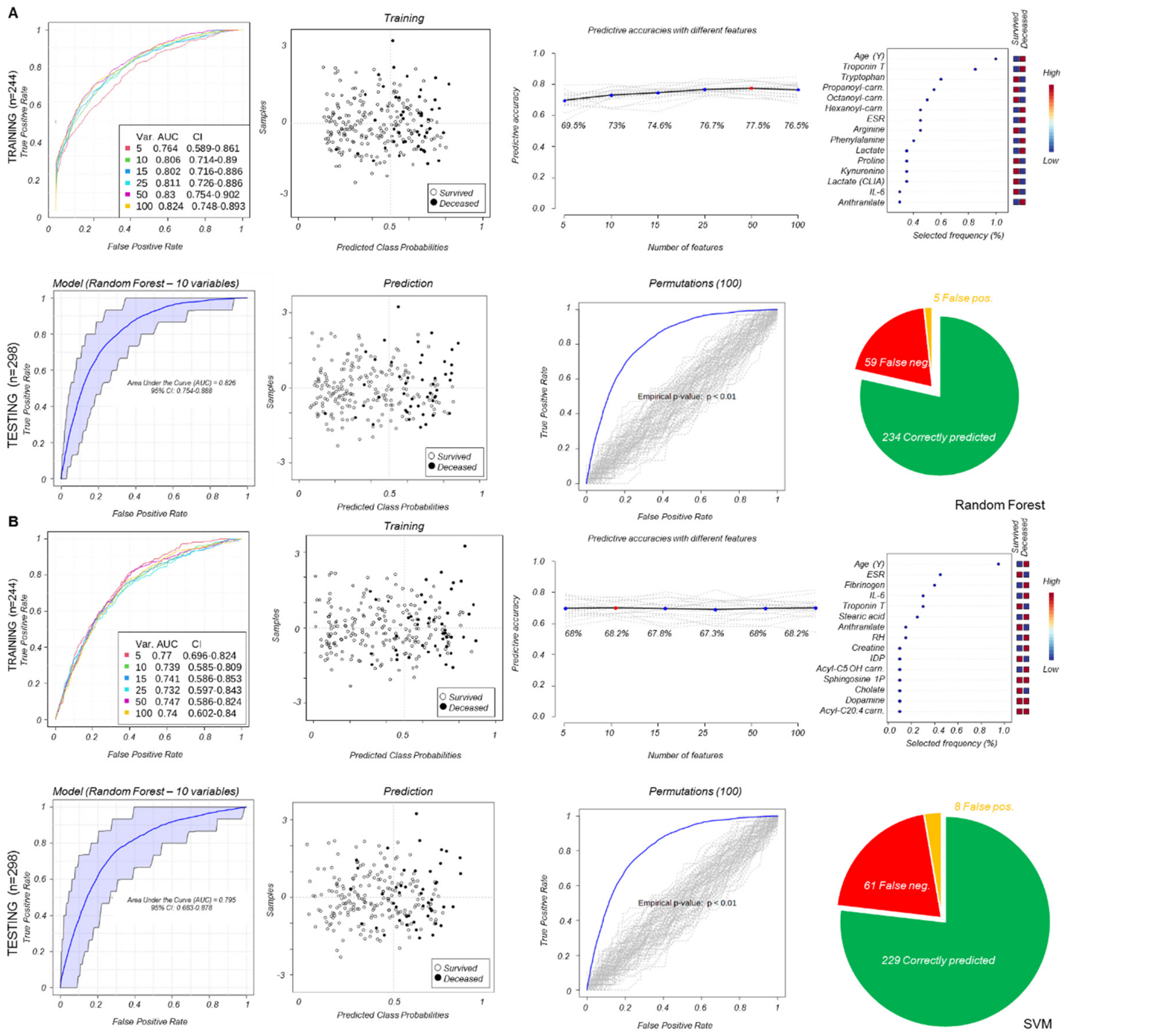

Supplementary Figure S10– Comparison of predictive models of mortality via Random Forest (A) and SVM (B) algorithms. Patients were curated for metabolomics, clinical, coagulation and inflammatory variables (total n = 542). The cohort was then divided in two groups, one for training (n = 244) and one for testing (n = 298) of the algorithm, which resulted to be ~78% and ~75% accurate, respectively.

Impact of being on ventilator

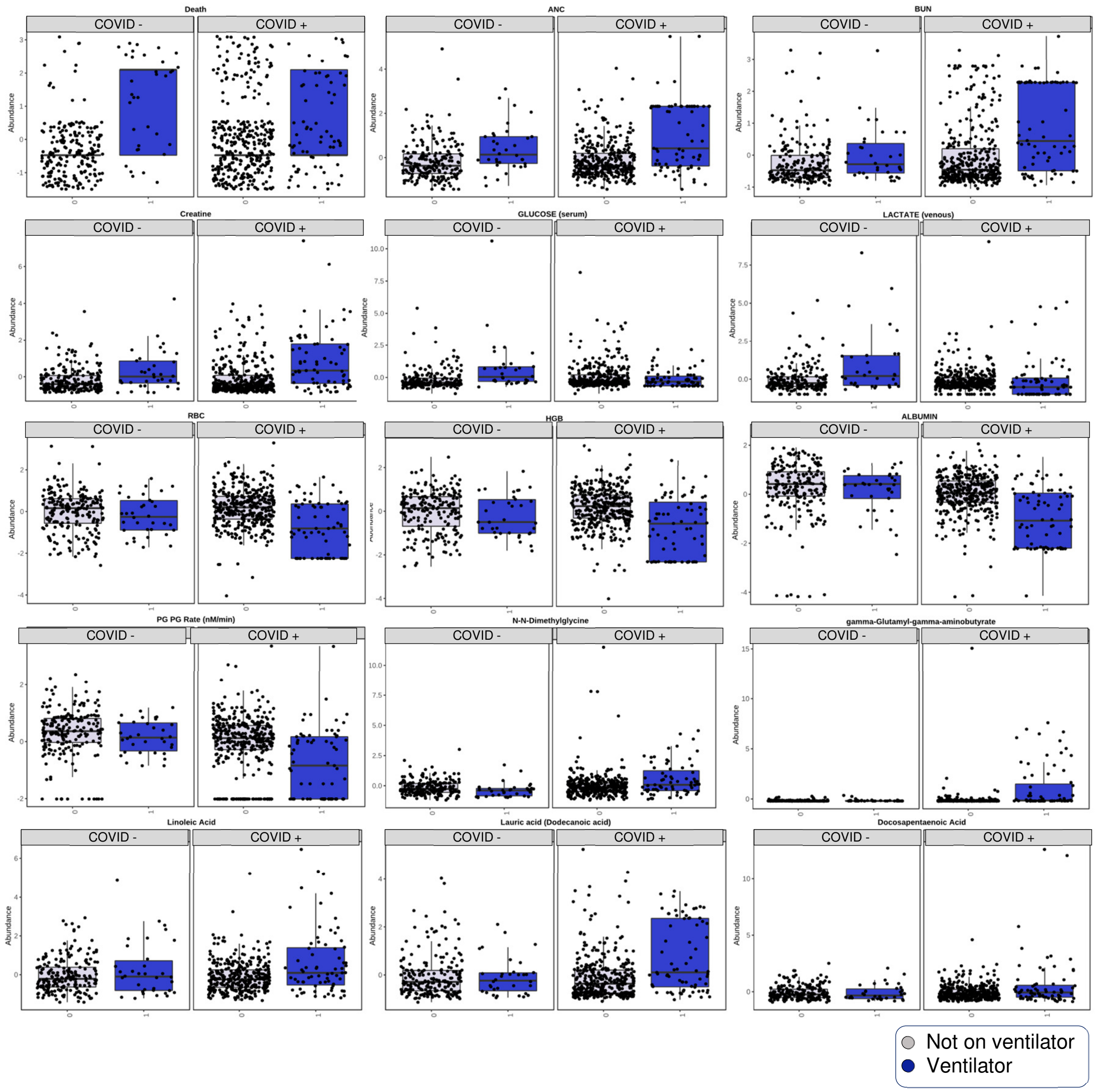

Supplementary Figure S11 – Markers of ventilators in COVID negative (-) and positive (+) patients, as determined by Two-way ANOVA (0 = not on ventilator; 1 = on ventilator).

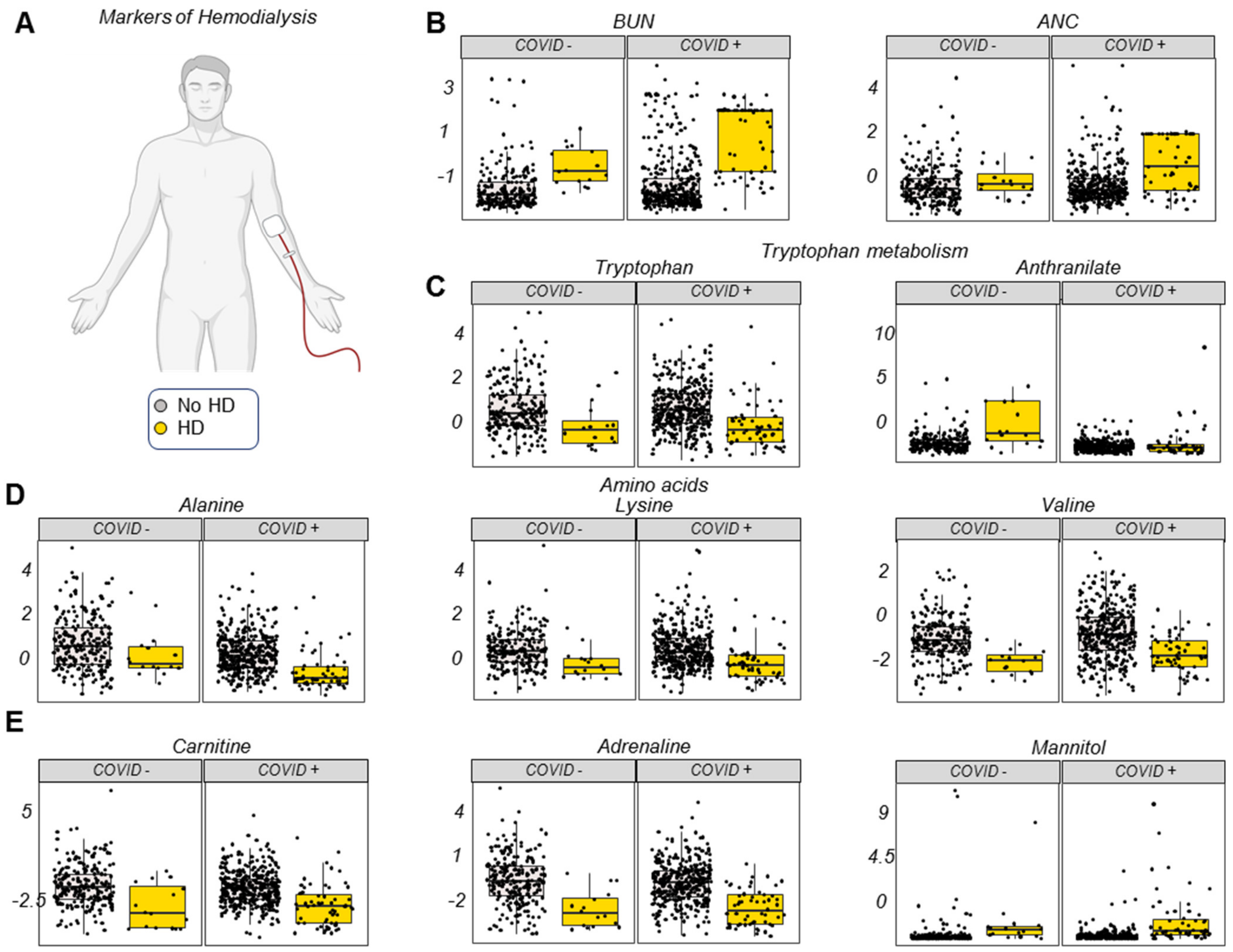

Supplementary Figure S12 – Markers of hemodialysis (without clotting) in COVID negative (-) and positive (+) patients, as determined by Two-way ANOVA (0 = no hemodialysis; 1 = on hemodialysis).

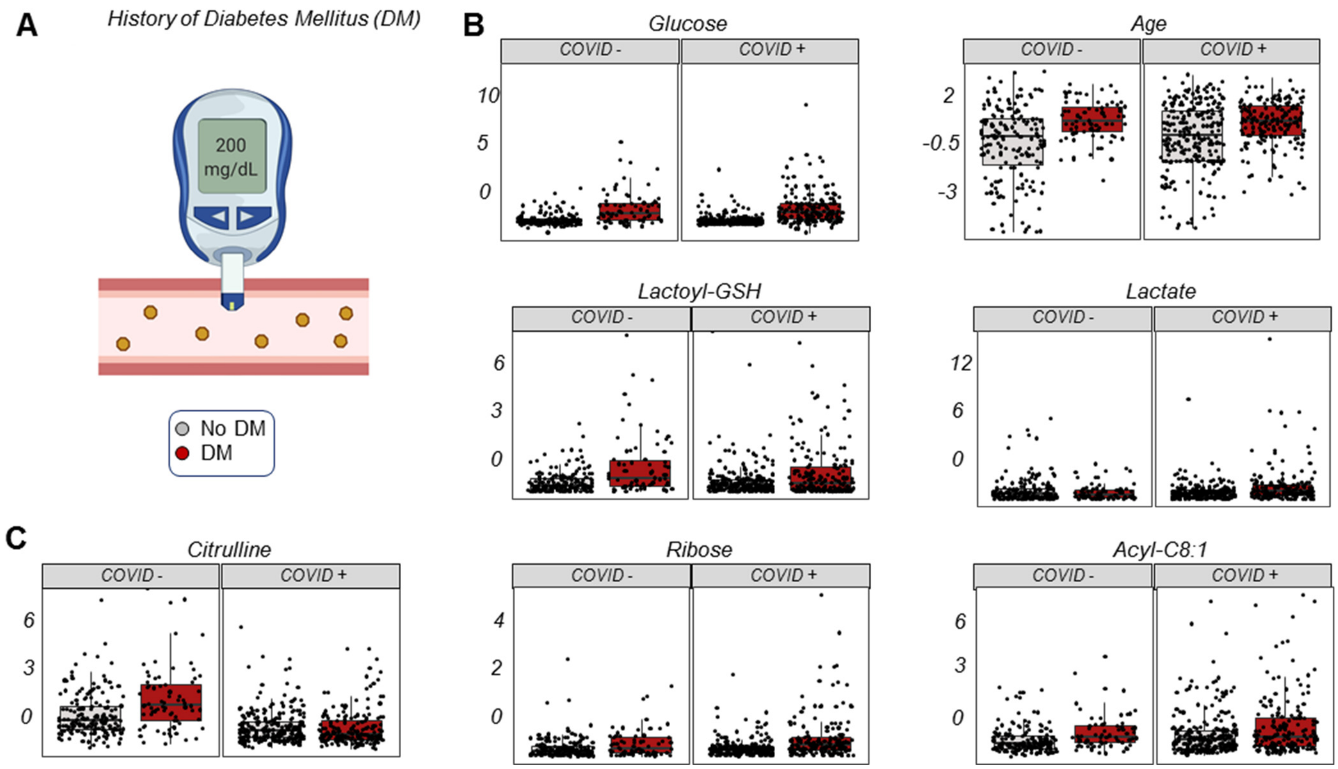

Supplementary Figure S13 – Impact of pre-existing conditions such as diabetes in COVID negative (-) and positive (+) patients, as determined by Two-way ANOVA (0 = no history of diabetes; 1 = diabetes).

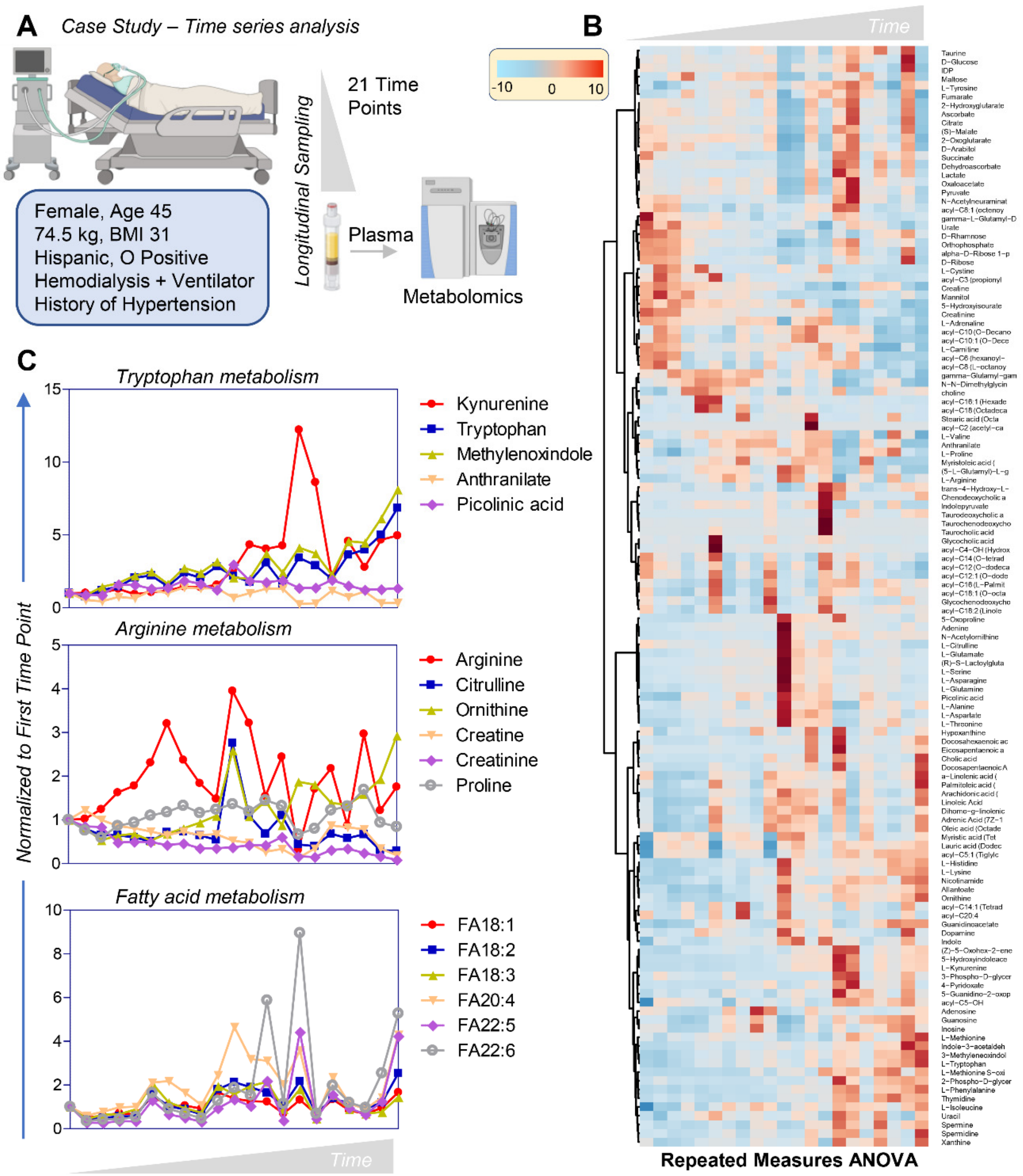

Supplementary Figure S14 – Time course analysis of a critically ill patient who survived.

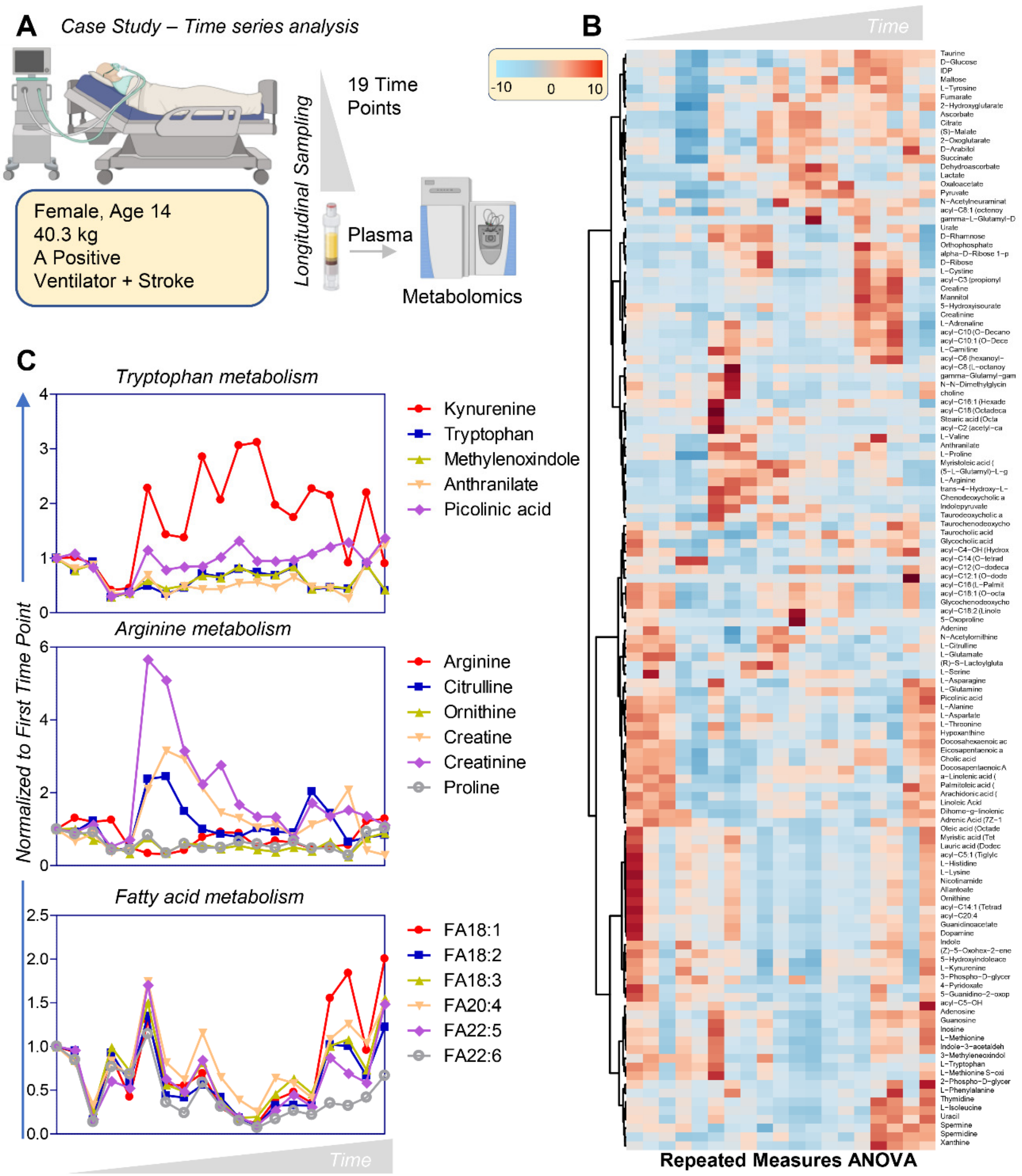

Supplementary Figure S15 – Time course analysis of a critically ill patient who survived (vectorial version of in manucript figure).

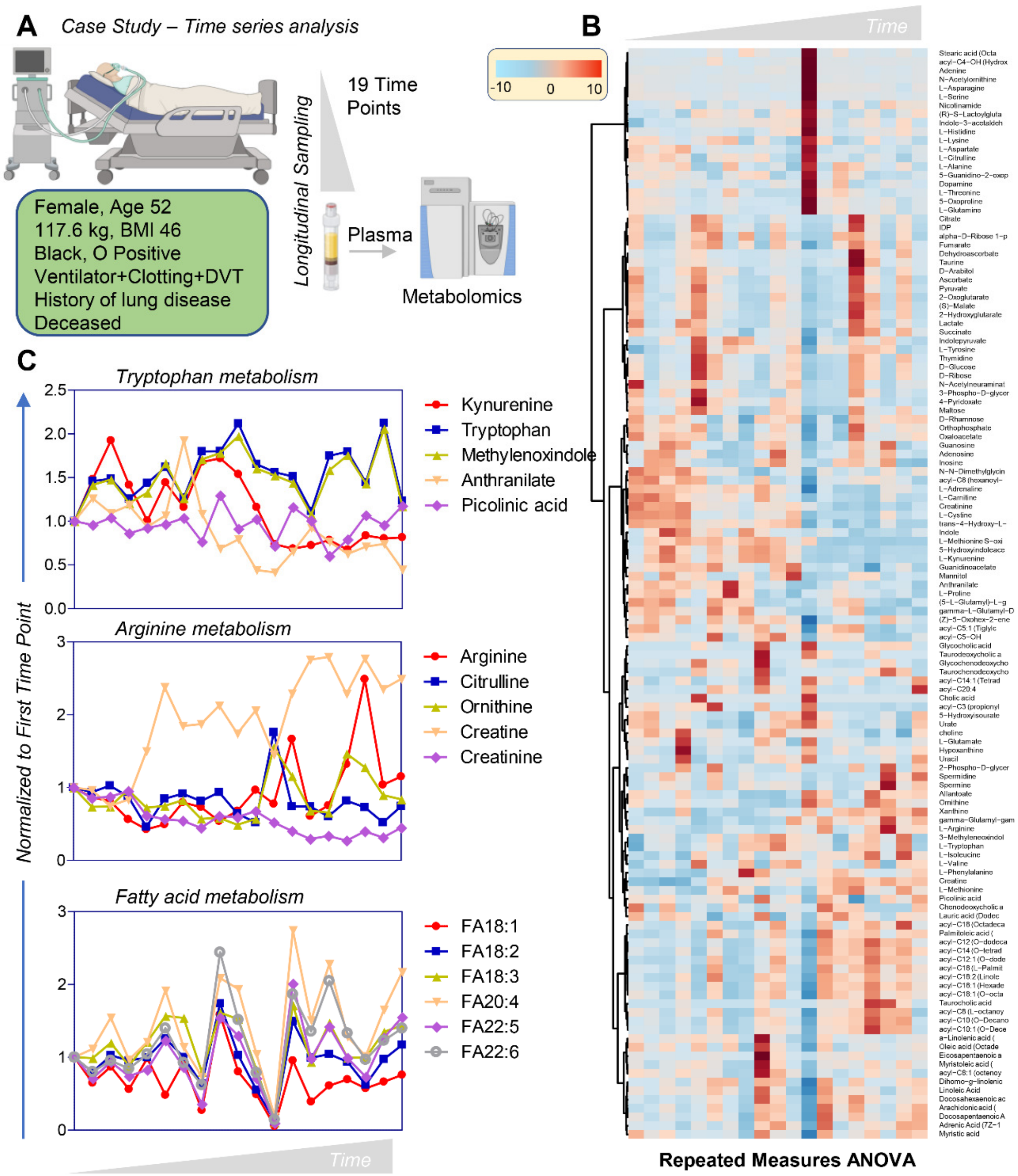

Supplementary Figure S16 – Time course analysis of a critically ill patient who died (vectorial version of in manucript figure).
